# Risk stratification and pathway analysis based on graph neural network and interpretable algorithm

**DOI:** 10.1186/s12859-022-04950-1

**Published:** 2022-09-27

**Authors:** Bilin Liang, Haifan Gong, Lu Lu, Jie Xu

**Affiliations:** Shanghai Artificial Intelligence Laboratory, Yunjing Road 701, Shanghai, China

**Keywords:** Graph neural network, Deep learning, Risk classification, Pathway, Interpretability

## Abstract

**Background:**

Pathway-based analysis of transcriptomic data has shown greater stability and better performance than traditional gene-based analysis. Until now, some pathway-based deep learning models have been developed for bioinformatic analysis, but these models have not fully considered the topological features of pathways, which limits the performance of the final prediction result.

**Results:**

To address this issue, we propose a novel model, called PathGNN, which constructs a Graph Neural Networks (GNNs) model that can capture topological features of pathways. As a case, PathGNN was applied to predict long-term survival of four types of cancer and achieved promising predictive performance when compared to other common methods. Furthermore, the adoption of an interpretation algorithm enabled the identification of plausible pathways associated with survival.

**Conclusion:**

PathGNN demonstrates that GNN can be effectively applied to build a pathway-based model, resulting in promising predictive power.

**Supplementary Information:**

The online version contains supplementary material available at 10.1186/s12859-022-04950-1.

## Abstract

Identification of gene expression profiles that discriminate disease phenotypes is a relatively routine research procedure in bioinformatics. Most traditional methods aim to discover gene-sets associated with disease phenotypes [1]. However, one of the limitations of clinical implementation is that those gene-sets identified by independent studies rarely display substantial overlap. For example, only 3 genes overlapped between the 70 and 76 genes that found significant association with metastatic breast cancer, respectively [2].

Pathway-based analysis of gene expression profiles has shown recently greater stability of biological activities and better performance [3]. The cancer samples were classified into multiple subtypes with distinct pathways that had different sensitivity to specific metabolic inhibitors [4, 5]. Another study reported that glioblastoma could be stratified into four functional subgroups based on pathway interference [6]. Moreover, some deep learning models had also been developed for pathway analysis or predicting clinical phenotypes, which is showing an advanced performance. For example, DeepOmix was applied in cancer survival analysis [7]; PACL was proposed for molecular subtyping of cancer [8]; a deep neural network was presented to predict the drug sensitivity [9]. Of note, a pathway-guided deep neural network (PGDNN) algorithm architecture has been usually adopted in those works. Generally, PGDNN consists of a gene layer (an input layer), a pathway layer, multi-hidden layers, and an output layer. Although PGDNN has shown promising performance, it cannot effectively capture the topological information hidden in pathway due to the limitation of algorithm architecture, which is important for bioinformatics analysis [10].

Recent advancements in graph neural networks (GNNs) have expand the capabilities to capture the hidden topological information in pathway. GNNs, a class of deep learning methods, has been designed to perform analysis on non-Euclidean data that was represented by graph consisting of nodes and relationships between edges [11]. Due to its ability to capture topological feature, GNNs have greater capabilities than deep neural networks (DNNs) and convolutional neural networks (CNNs) on graph dataset [12]. Recently, GNNs have been successfully introduced to the recommendation system, computer vision and chemistry [13]. Additionally, GNNs have been gradually adopted in recent bioinformatics research. For example, a GNNs model was proposed for cancer gene prediction across different cancer types [14]; the breast cancer subtypes were identified through a GNNs model analyzing the protein–protein interactions network [15].

Furthermore, interpretation algorithms for deep learning model generally are often used for biological interpretation, such as identifying pathways associated with disease phenotypes. Until now, various strategies have been proposed to interpret deep learning models. Recently, the integrated gradients (IG) algorithm has been widely used for interpretation in community [16]. IG measures feature attribution through aggregating gradients along a straight-line path between input sample and the baseline [17]. Compared to other algorithms, IG involves two theoretical advantages: the first is sensitivity; the second is implementation invariance, which states that regardless of the model architecture, if the models are functionally equivalent (the same output from any input), then their feature attributions will also be equivalent [17]. IG has been applied to multiple biological studies, such as assisting grading for diabetic retinopathy [18], interpretable visualization of retrosynthetic reactions [19] and improving the detection of lymph node metastases in breast cancer patients [20]. Comparatively, however, few studies have adopted IG to explain deep learning model building with gene expression profiles to detect potential mechanisms associated with phenotypes.

In this study, we proposed a novel method, called PathGNN, based on the hypothesis that GNNs can effectively capture the topological feature of pathway, thereby improving model performance. In addition, we introduced IG interpretation algorithms to explain PathGNN model for identifying key pathways associated disease phenotypes. As a case, we performed PathGNN to predict cancer survival and detect plausible pathways using transcriptomic and clinical data, which is a fundamentally challenging problem in bioinformatics. We demonstrated the method achieved promising predictive performance in differentiating between long-term survival (LTS) and non-LTS when applied to multiple cancers. Through the proposed methods, we show that the resulting GNN model achieves much better predictive performance due to capturing the topological information hidden in pathway.

## Materials and methods

### Gene expression and clinical datasets

The gene expression matrix and clinical data of cancer patients were download from TCGA [21]. Individuals with missing clinical or gene expression data were excluded. Long-term survival (LTS) was defined as survival > $k$ years after diagnosis, whereas non-LTS was defined as survival < = $k$ years ($k\in [\mathrm{1,3},\mathrm{5,10}]$). The gene expression matrix is denoted as $\mathrm{M}\in {\mathrm{R}}^{\mathrm{n}\times \mathrm{r}}$, where n and r indicate the number of samples and genes, respectively, and the value in the matrix is FPKM (Fragments per Kilobase of exon model per Million mapped fragments) that quantifies the gene expression.

After data preprocessing, we selected four types of cancer (i.e., lung adenocarcinoma, LUAD, N = 515; kidney renal clear cell carcinoma, KIRC, N = 530; low grade glioma, LGG, N = 511; skin cutaneous melanoma, SKCM, N = 468). We determined the survival years threshold k = 3 to validate our method, which was considered to have a larger data sample size and better data balance. After removing individuals who missed clinical information or survived but had their last followed-up ≤ 3 years, the LTS and non-LTS groups had 132 and 137, 232 and 110, 155 and 75, 290 and 111 cases for LUAD, SKCM, LGG and KIRC, respectively. Age and tumor stage were used as clinical features through feature selection.

### Graph construction with gene expression and pathway network data

Typically, a graph is defined as $\mathcal{G}=(v, e)$, where $v$ is a set of nodes and $e$ is the edges between them. We proposed to construct pathway graph: genes as nodes $v$; metabolite and protein-mediated gene interactions [22] as edges $e$ and gene expression data as node’s feature $x$. To construct the graph, pathway and gene network information was retrieved from the Reactome database [23] using graphite [22], a R package for automatic download pathway information and converting pathway to graph. Then, we removed pathways with less than 15 genes or more than 400 genes and duplicated pathways.

**Fig. 1:**
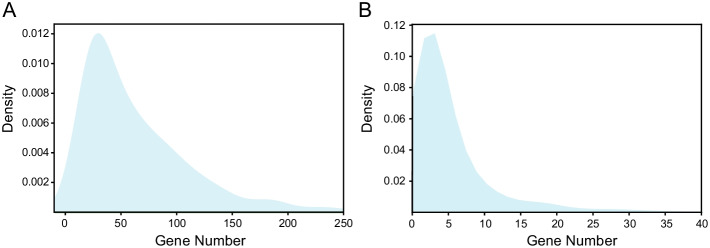
The visualization of pathways and clinical features in this study. (**A**) The density distribution of the gene number in each pathway. (**B**) The density distribution of the number of each gene shared between pathways

In total, 2390 pathways downloaded from Reactome database, while 855 pathways were remained for followed analysis (Additional file 1). There were 8611 unique genes involved in those 855 pathways, with a median of 50 genes in each pathway (Fig. 1A). We counted the sharing of genes among pathways, and the result showed that 1613 genes involved unique pathway, 50% genes shared less than 3 pathways, 95% genes shared less than 22 pathways and the max shared pathways number was 275 (Fig. 1B).

### PathGNN architecture

**Fig. 2:**
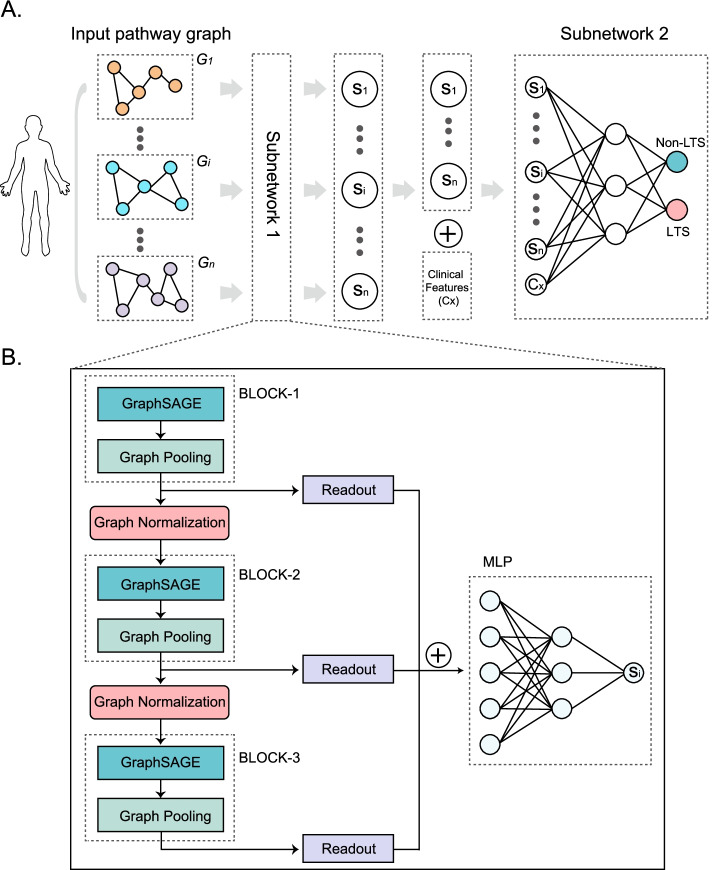
The deep learning framework implemented in PathGNN. (**A**) The overview of deep learning framework in PathGNN. PathGNN was designed as two components, named Subnetwork1 and Subnetwork2, respectively. (**B**) The schematic view of Subnetwork1 architecture

An overview of the proposed PathGNN model was shown in Fig. 2A. We considered clinical outcome being associated with pathway abnormalities and clinical features. Feature selection was performed on clinical features by statistical tests (t-test or chi-square test). PathGNN was designed as two components, named Subnetwork1 and Subnetwork2, respectively. The Subnetwork1 was used to capture features hidden in pathways; the Subnetwork2 classified patients into LTS and non-LTS.

The architecture of the Subnetwork1 is comprised of three blocks (i.e., BLOCK-1/2/3), a readout layer after each block (i.e., Readout), graph normalization layer between blocks (i.e., Graph Normalization) and multilayer perceptrons (i.e., MLP) (Fig. 2B). Among them, each block is used to learn the feature representation of the pathway, which includes a graph convolution layer and a graph pooling layer.

A more detailed description is shown in pseudocode, all pathway graphs ($G$) are input in Subnetwork1, and then each graph (${g}_{i}$, $i$ represents $i$-th graph) is subjected to multiple graph neural network blocks ($N$). In each block, 3 layers are performed on the graph, as follow: (1) feature representation learning is first performed on the pathway graph using GraphSAGE [24]. GraphSAGE is used to generate node embeddings by sampling and aggregating feature information from local neighborhoods, which is suitable for overcoming inductive bias, since a patient includes hundreds of graphs. (2) As the number of pathways and genes varies greatly in the pathway graph, thus hierarchical representation is needed to make the model adaptive to the different number of nodes. Thus, the graph pooling layer is implemented by SAGPool algorithm [25] to yield hierarchical representations. (3) The readout layer is designed to compute a feature vector of the whole graph using Set2Set algorithm [26] based on iterative content-based attention.

There is a graph normalization layer after each block, which has been demonstrated to be effective for GNNs with multiple layers [27]. Finally, let $H=[{h}_{1}, {h}_{2},{h}_{3}]$ be the input of MLP, where ${h}_{1}, {h}_{2},{h}_{3}$ are the output of Readout layers. Using a MLP module with two neural network layers to calculate ${S}_{i}$, as follow:

$$hidden= \sigma \left({W}_{1}\bullet H + {b}_{1}\right)$$

$${S}_{i} = \sigma \left({W}_{2}\bullet hidden + c\right)$$

where $\upsigma$ is the $tanh$ activation function, ${W}_{1}$ and ${W}_{2}$ are the weight matrix, ${b}_{1}$ and $c$ are the bias term, ${S}_{i}$ represents the comprehensive features of $ith$ pathway extracted by Subnetwork1.


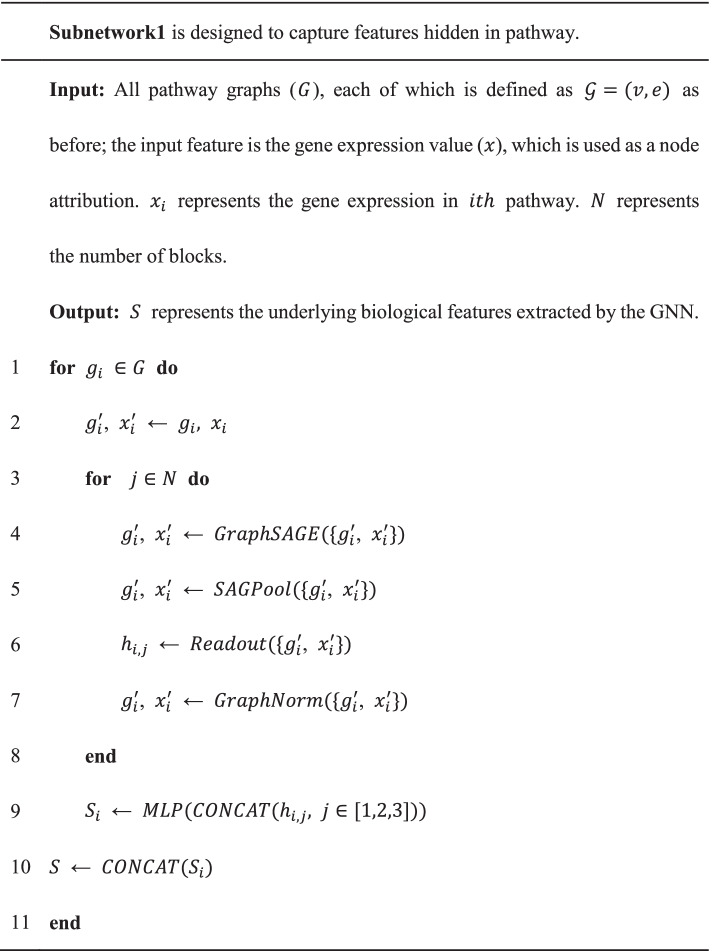


The representations ($S$) output from the Subnetwork1 indicates the comprehensive feature including topological and gene-sets information in pathways. After this, we designed Subnetwork2 for predicting clinical outcome, in which both $S$ and clinical features are concatenated as input data (Fig. 2A). More specifically, the Subnetwork2 is built based on MLP that consisted of an input layer, two hidden layers with 128 and 32 nodes, respectively, a dropout layer with a dropout rate of 40% and an output layer. Each hidden layer was immediately followed by a rectified linear unit (ReLU) activation function. Finally, we use the cross-entropy loss and fivefold cross-validation (CV) to train the model.

### Training the PathGNN model

The models are built based on Pytorch (version, 1.8) [28] and Pytorch Geometric (version, 2.0.3) [29]. We trained the model for 250 epochs, and the batch size was set to 60. Adam was used to optimizing the model, with a learning rate of 0.001 for the first 150 epochs and 0.0005 for the last 100 epochs. We evaluated the performance of the model with the area under the curve (AUC) metric. We trained the models with NVIDIA Tesla V100 GPU with 32 GB memory.

### Ablation study

To understand the contribution of the component to the overall system, an optimization study by removing or replacing individual modules was conducted on LUAD. Specifically, we first replaced the GraphSAGE with graph attention network (GAT) or graph convolutional network (GCN) to investigate the impact on applying different graph convolution algorithm. Next, we removed or added blocks in the Subnetwork1 to explore the effect of different number of blocks. In addition, the Set2Set that adopted in the PathGNN architecture is a global pooling algorithm based on iterative content attention. We replaced the Set2Set with global add pooling and global average pooling algorithm, which returns graph-level outputs by adding and averaging node features across the node dimension, respectively. The experimental training procedure for this method optimization was the same as before.

### Biological interpretation using integrated gradient

The aberrant pathways associated with clinical prognosis were identified by IG. Here, we calculated the importance value (${IG score}_{i}$) for the $ith$ pathway as follow:

$${IG score}_{i}=\left({S}_{i}- {S}_{i}^{\mathrm{^{\prime}}}\right){\int }_{0}^{1}\frac{\partial f\left({S}_{i}^{\mathrm{^{\prime}}} + \alpha \left(\left({S}_{i}- {S}_{i}^{\mathrm{^{\prime}}}\right)\right)\right)}{{\partial S}_{i}^{\mathrm{^{\prime}}}}d\alpha$$

where ${S}_{i}$ denotes the input feature that output from Subnetwork1, $f$ represents the neural network model (i.e., PathGNN model) and ${S}_{i}^{^{\prime}}$ represents the baseline. The subscript $i$ is the index of $ith$ pathway.

The IG scores were z-transformed and those pathways with z-score value (${IG score}_{i}^{z}$) greater 1.96 (i.e., *P value* < 0.05) [30] were considered as candidate aberrant pathways.

### Comparison with benchmark methods

The predictive performance of PathGNN was compared with several machine learning methods, including random forest (RF), deep neural network (DNN), logistic regression (LR) and pathway-guided deep neutral network (PGDNN) [7–9]. For all experiments, the same experimental settings were used as described in the above cross validation section. RF and LR were performed using scikit-learn package (version, 1.0.1) [31], while DNN and PGDNN were built by Pytorch.

The DNN model was constructed with four hidden layers, where the number of nodes were 855, 128, 64 and 16, respectively. For PGDNN, the architecture was the same as DNN, but the first hidden layer, named pathway layer, which represents the biological pathways linked with input genes. The construction of these two models can be found in the Additional file 2. 

## Result

### Ablation study

**Fig. 3:**
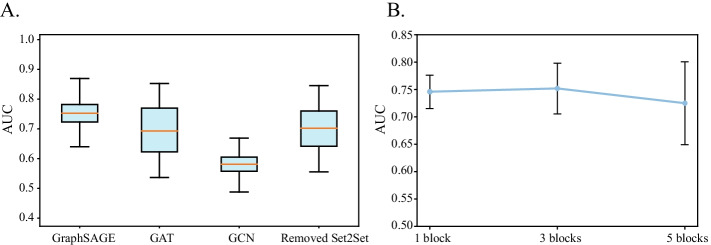
The results of ablation study. (**A**) “GraphSAGE, GAT and GCN” indicated the model results of the corresponding graph convolution algorithm used; “Removed Set2Set” indicated that GraphSAGE was used as the convolution algorithm, but the Set2Set layer was removed. (**B**) The model performance with different number of blocks

The result of architecture optimization was shown in Fig. 3. First, we investigated the impact on replacing the graph convolution algorithm (Fig. 3A). When we replaced GraphSAGE with GAT or GCN, the overall performance would drop by about 6 and 16%, respectively. This comparison demonstrates that GraphSAGE is more effective at dealing with biological pathways. Next, we compared the performance when setting 1, 3 and 5 blocks in Subnetwork1. The results showed that the model performs better when the number of blocks was set to 3 (Fig. 3B). In addition, after replacing Set2Set with global add pooling and global average pooling algorithm (Fig. 3A), the overall performance would drop by about 5%, which suggested attention mechanism is more effective at capturing the features of pathways.

### Modeling performance and comparison with benchmark methods

The PathGNN model was trained to classify LTS and non-LTS groups in multiple cancers. In addition, the predictive performance was compared with four different machine learning methods, including logistic regression, random forest, DNN and PGDNN. Those methods were constructed as described in the methods section. As a result, the predictive performance for five algorithms on four datasets mentioned above were summarized in Table 1. The PathGNN shown average AUCs of 0.752, 0.702, 0.862 and 0.754 in LUAD, SKCM, LGG and KIRC, respectively. Of note, the predictive performance of PathGNN showed significantly better than other machine learning methods on four cancer datasets, especially on LUAD, SKCM and KIRC. It should be noted that PGDNN received better performance than DNN, logistic regression and random forest, which suggested that pathway-based deep learning model has better performance for predicting clinical phenotypes and prognosis (Additional file 2: Table 1).

**Table 1:** Comparison of predictive performance with benchmark methods

Cancer	PathGNN	PGDNN	DNN	RF	LR
LUAD	**0.752 ± 0.046**	0.592 ± 0.026	0.536 ± 0.059	0.566 ± 0.043	0.586 ± 0.057
SKCM	**0.702 ± 0.041**	0.626 ± 0.035	0.590 ± 0.031	0.601 ± 0.055	0.588 ± 0.056
LGG	**0.862 ± 0.058**	0.852 ± 0.082	0.834 ± 0.080	0.808 ± 0.083	0.755 ± 0.068
KIRC	**0.754 ± 0.071**	0.683 ± 0.045	0.584 ± 0.088	0.608 ± 0.042	0.604 ± 0.027

The predictive performance of PathGNN was compared with pathway-guided deep neutral network (PGDNN), deep neural network (DNN), random forest (RF) and logistic regression (LR). The area under the curve (AUC: mean ± standard deviation) was recorded

Bold indicated the best predictive performance in each case study

### Identification of key pathways associated with survival

To identify the biological mechanisms associated with survival in multiple cancer patients and independent from clinical variables, the clinical features pathological stage and age were removed from the model. As a result of IG, 13, 4, 9 and 5 pathways were finally identified in LUAD, SKCM, LGG and KIRC, respectively (z score > 1.96; *P value* < 0.05) (Additional file 3: Table 2). Among them, some key pathways are clearly associated with cancer progression and prognosis (Table 2). In the result of LUAD, two aberrant regulation of mitotic cell cycle due to RB1 pathways, a RUNX3 expression and activity pathway were found. A ERBB4 signaling pathway was identified in SKCM. An aberrant regulation of mitotic cell cycle due to RB1 pathway, a FGFR signaling pathway and a p53 pathway were found in KIRC. An aberrant regulation of mitotic cell cycle due to RB1 pathway and the p53 pathway were detected in LGG. Additionally, the aberrant regulation of mitotic cell cycle due to RB1 pathway was recurred between LUAD, KIRC and LGG; the sialic acid metabolism was shared between LUAD and KIRC.

**Table 2:** Key pathways associated with the long-term survival in multiple cancers

Pathway	Cancer	IG score^z^
Aberrant regulation of mitotic cell cycle due to RB1 defects	LUAD	2.161
Aberrant regulation of mitotic G1/S transition in cancer due to RB1 defects	LUAD	3.153
E3 ubiquitin ligases ubiquitinate target proteins	LUAD	3.100
Regulation of RUNX3 expression and activity	LUAD	2.372
Signaling by ERBB4	SKCM	3.628
Aberrant regulation of mitotic G1/S transition in cancer due to RB1 defects	KIRC	3.523
Signaling by FGFR	KIRC	3.525
Stabilization of p53	KIRC	1.970
Aberrant regulation of mitotic G1/S transition in cancer due to RB1 defects	LGG	4.955
Regulation of TP53 Expression and Degradation	LGG	3.927

IG score^z^ indicated the importance of the pathway in predicting clinical risk classification, and the larger the value the higher the importance

**Fig. 4:**
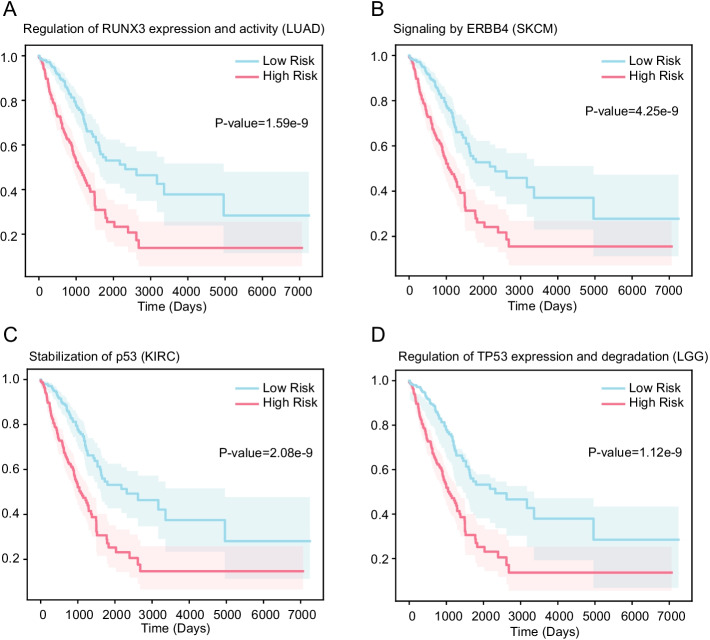
Kaplan–Meier curves for two groups dichotomized by a median split in IG scores. The shaded area represents the 95% confidence interval

A Kaplan–Meier analysis was performed to compared survival time between two groups dichotomized by a median split of IG score in the key pathways. That is, given a key pathway associated with disease phenotypes, IG scores across all specific cancer samples were dichotomized by a median split. Then, the log-rank test was performed for each key pathway. As shown in Additional file 3: Table 2, 30 of 31 pathways showed statistically significant difference between two groups (*P value* < 0.05). The survival curves of partial pathways that have been demonstrated to be associated with cancer prognosis were shown in Fig. 4, and the survival time was statistically significant between two groups.

## Discussion

Pathway-based analysis of transcriptomic data has shown recently greater stability of biological activities and better performance than gene-based analysis [3]. Until now, this analysis strategy has been applied in biomedical research, such as identification of disease subtypes [3, 5], predicting prognosis of complex disease [32] and predicting drug response [9]. Moreover, some pathway-based deep learning models have been also developed recently, such as DeepOmix [7] and PACL [8]. In terms of methodology, a PGDNN deep learning architecture has been utilized in those studies. Generally, PGDNN architecture consists of a gene layer (an input layer), a pathway layer, multi-hidden layers, and an output layer. The pathway layer is a partially connected layer based on the biological pathways linked with input genes. PGDNN architecture has shown better performance than other early machine learning methods due to capturing the gene set information in pathway. However, it lacks the topological information of genes interconnection in pathway, which is important for biological process [10].

The pathway is considered as a set of genes and their interactions with specific biological function, which is a graph data structure. In addition, GNNs are a class of deep learning methods that show better performance on graph than DNNs, CNNs or other machine learning methods due to the ability to capture topological information. Therefore, we proposed a novel deep learning method based on GNNs, called PathGNN, which has the additional ability to capture the topological information hidden in pathway.

We applied PathGNN to predict 3-year survival of four types of cancer (i.e., LUAD, SKCM, LGG and KIRC) using transcriptomic and clinical data, considering that the sample size of LTS and non-LTS was relatively balanced, which was conductive to verifying our method. The predictive performance of PathGNN in differentiating between LTS and non-LTS shown an average AUCs of 0.723, 0.702, 0.862 and 0.754 were obtained in LUAD, SKCM, LGG and KIRC, respectively (Table 1). Overall, PathGNN significantly improved predictive performance compared to the other four methods. Specifically, PathGNN and PGDNN achieved greater AUCs in all types of cancer than DNN, RF and LR, suggesting that pathway information can assist to improve predictive performance, which is consistent with prior studies [7, 9, 33]. Besides this, PathGNN also achieved better performance than PGDNN, indicating that deep learning models are expected to gain further predictive power if the topological features hidden in pathways can be captured.

The IG algorithm was utilized in PathGNN to detect those pathways that associated with clinical outcomes. There were 13, 4, 9 and 5 key pathways identified on LUAD, SKCM, LGG and KIRC, respectively (Additional file 3: Table 2). Among them, 17 pathways were supported by related works (7 for LUAD, 2 for SKCM, 4 for LGG and 4 for KIRC) (Additional file 3: Table 2). The negative pathways were mainly associated with biological events, such as muscle contraction. The positive pathways were mainly related to specific regulatory mechanisms, such as signaling by FGFR. Some of the positive pathways were associated with cancer as follows. The regulation of RUNX3 expression and activity pathway detected in LUAD has been reported to accelerate malignant progression of lung adenocarcinoma [34]. Two studies suggested that ERBB4 is disorder in melanoma, and ERBB4 kinase inhibition may constitute an effective therapeutic strategy [35, 36]. For LGG, a recent study showed TP53 is key driver of lower grade glioma therapeutic efficacy and influence survival outcomes [37]. For KIRC, fibroblast growth factor receptor (FGFR) pathway is involved tumor angiogenesis and escaping vascular endothelial growth factor (VEGF)-targeted therapies [38]. Of note, the aberrant regulation of mitotic cell cycle due to RB1 defects pathway was identified in LUAD, KIRC and LGG. According to prior study, irreversible defects in RB1 tumor suppressor functions often predict poor outcomes in many human cancers, such as lung adenocarcinoma [39].

In conclusion, our PathGNN includes two advantages: (1) it inherits the advantage of PGDNN due to the introduction of gene-sets in pathways into modeling; (2) the topological information of pathway is remained through GNNs. In this study, PathGNN achieved further predictive power than PGDNN and the other machine learning methods, which suggested topological feature can improve model performance based on pathway. In short, PathGNN is expected to further promote the application of pathway-based analysis in biomedical field.

The inherent limitation of our approach is that it can identify key pathways with disease phenotype, but not how the pathway activation trend affects disease phenotype. This limitation could be extended in a future extension through transfer learning.

## Supplementary Information

**Additional file 1**. The information of total pathways for building PathGNN model.

**Additional file 2**. The architecture of DNN and PGDNN.

**Additional file 3**. The key pathways associated with cancer survival identified by PathGNN.

## Data Availability

The source code and dataset are freely available at: https://github.com/BioAI-kits/PathGNN.

## Abbreviations

GNNs: Graph neural networks
LTS: Long-term survival
PGDNN: Pathway-guided deep neural network
CNNs: Convolutional neural networks
IG: Integrated gradients
RF: Random forest
LR: Logistic regression
LUAD: Lung adenocarcinoma
KIRC: Kidney renal clear cell carcinoma
LGG: Low grade glioma
GBM: Glioblastoma multiforme
