# Supplementary material for: Risk stratification and pathway analysis based on graph neural network and interpretable algorithm
Source: BMC Bioinformatics. 2022 Sep 27;23:394. doi: 10.1186/s12859-022-04950-1 (PMC9516820; doi:10.1186/s12859-022-04950-1)
Supplement: Supplementary file 1 — Additional file 1. The information of total pathways for building PathGNN model. [file 12859_2022_4950_MOESM1_ESM.docx]

**Additional file 1** | This table includes the information of total pathways for building PathGNN model. All pathways download from Reactome dataset. “Native ID” represents the ID in dataset; “Nodes” represents the gene number in each pathway; “Edges” represents the edge number in each pathway.

| **Pathway** | **Native ID** | **Nodes** | **Edges** |
| --- | --- | --- | --- |
| Diseases of metabolism | R-HSA-5668914 | 394 | 724 |
| Metabolism of vitamins and cofactors | R-HSA-196854 | 375 | 3139 |
| DNA Repair | R-HSA-73894 | 362 | 11215 |
| MAPK family signaling cascades | R-HSA-5683057 | 351 | 5894 |
| Intracellular signaling by second messengers | R-HSA-9006925 | 333 | 4762 |
| Translation | R-HSA-72766 | 329 | 16833 |
| Signaling by WNT | R-HSA-195721 | 323 | 6525 |
| Signaling by Nuclear Receptors | R-HSA-9006931 | 320 | 3819 |
| Transmission across Chemical Synapses | R-HSA-112315 | 319 | 5680 |
| Phospholipid metabolism | R-HSA-1483257 | 315 | 3961 |
| Antigen processing: Ubiquitination & Proteasome degradation | R-HSA-983168 | 315 | 22519 |
| Extracellular matrix organization | R-HSA-1474244 | 312 | 3696 |
| ERK1/ERK2 pathway | R-HSA-5684996 | 312 | 5462 |
| RAF/MAP kinase cascade | R-HSA-5673001 | 306 | 5406 |
| RHO GTPase Effectors | R-HSA-195258 | 302 | 8245 |
| Metabolism of steroids | R-HSA-8957322 | 301 | 3396 |
| Organelle biogenesis and maintenance | R-HSA-1852241 | 301 | 6923 |
| Phase I - Functionalization of compounds | R-HSA-211945 | 297 | 4155 |
| Anti-inflammatory response favouring Leishmania parasite infection | R-HSA-9662851 | 294 | 9592 |
| Interferon Signaling | R-HSA-913531 | 291 | 2554 |
| Deubiquitination | R-HSA-5688426 | 288 | 3140 |
| PI3K/AKT Signaling | R-HSA-1257604 | 286 | 4256 |
| G alpha (q) signalling events | R-HSA-416476 | 282 | 5786 |
| Cell Cycle Checkpoints | R-HSA-69620 | 278 | 7827 |
| Disorders of transmembrane transporters | R-HSA-5619115 | 269 | 2263 |
| Phase II - Conjugation of compounds | R-HSA-156580 | 258 | 1545 |
| HIV Infection | R-HSA-162906 | 257 | 8464 |
| Chromatin modifying enzymes | R-HSA-3247509 | 256 | 4024 |
| Metabolism of water-soluble vitamins and cofactors | R-HSA-196849 | 252 | 1620 |
| Cell surface interactions at the vascular wall | R-HSA-202733 | 251 | 2293 |
| Nucleotide metabolism | R-HSA-15869 | 250 | 3421 |
| Mitotic Metaphase and Anaphase | R-HSA-2555396 | 248 | 8742 |
| Mitotic Anaphase | R-HSA-68882 | 247 | 8736 |
| HCMV Infection | R-HSA-9609646 | 244 | 13713 |
| Processing of Capped Intron-Containing Pre-mRNA | R-HSA-72203 | 244 | 22021 |
| Neddylation | R-HSA-8951664 | 239 | 6955 |
| The citric acid (TCA) cycle and respiratory electron transport | R-HSA-1428517 | 234 | 5479 |
| Fc epsilon receptor (FCERI) signaling | R-HSA-2454202 | 234 | 6511 |
| Programmed Cell Death | R-HSA-5357801 | 230 | 1919 |
| Signaling by ROBO receptors | R-HSA-376176 | 226 | 7527 |
| ESR-mediated signaling | R-HSA-8939211 | 223 | 2868 |
| Glycerophospholipid biosynthesis | R-HSA-1483206 | 222 | 2200 |
| Signaling by NOTCH | R-HSA-157118 | 218 | 3074 |
| Transcriptional regulation by RUNX1 | R-HSA-8878171 | 214 | 3973 |
| TCF dependent signaling in response to WNT | R-HSA-201681 | 213 | 4126 |
| rRNA processing | R-HSA-72312 | 211 | 8276 |
| Neurotransmitter receptors and postsynaptic signal transmission | R-HSA-112314 | 209 | 3432 |
| Muscle contraction | R-HSA-397014 | 208 | 5282 |
| Ub-specific processing proteases | R-HSA-5689880 | 206 | 2367 |
| Diseases of glycosylation | R-HSA-3781865 | 202 | 393 |
| Ion channel transport | R-HSA-983712 | 201 | 3049 |
| rRNA processing in the nucleus and cytosol | R-HSA-8868773 | 201 | 8255 |
| Transport to the Golgi and subsequent modification | R-HSA-948021 | 200 | 5302 |
| Cellular response to chemical stress | R-HSA-9711123 | 199 | 2519 |
| Intra-Golgi and retrograde Golgi-to-ER traffic | R-HSA-6811442 | 199 | 3901 |
| C-type lectin receptors (CLRs) | R-HSA-5621481 | 196 | 2202 |
| Antigen processing-Cross presentation | R-HSA-1236975 | 195 | 2335 |
| Mitotic Prometaphase | R-HSA-68877 | 194 | 9672 |
| Mitotic G2-G2/M phases | R-HSA-453274 | 193 | 6055 |
| Cilium Assembly | R-HSA-5617833 | 193 | 6352 |
| Fcgamma receptor (FCGR) dependent phagocytosis | R-HSA-2029480 | 193 | 6934 |
| G2/M Transition | R-HSA-69275 | 191 | 6039 |
| RAC1 GTPase cycle | R-HSA-9013149 | 190 | 784 |
| HCMV Early Events | R-HSA-9609690 | 189 | 6682 |
| B Cell Activation | R-HSA-983705 | 188 | 5387 |
| mRNA Splicing | R-HSA-72172 | 188 | 16984 |
| Apoptosis | R-HSA-109581 | 187 | 1669 |
| SUMOylation | R-HSA-2990846 | 187 | 1954 |
| Toll-like Receptor Cascades | R-HSA-168898 | 185 | 2065 |
| Cytochrome P450 - arranged by substrate type | R-HSA-211897 | 184 | 2429 |
| Major pathway of rRNA processing in the nucleolus and cytosol | R-HSA-6791226 | 183 | 7797 |
| pre-mRNA splicing | R-HSA-72163 | 180 | 16604 |
| Glycosaminoglycan metabolism | R-HSA-1630316 | 179 | 1415 |
| SUMO E3 ligases SUMOylate target proteins | R-HSA-3108232 | 178 | 1903 |
| S Phase | R-HSA-69242 | 178 | 3270 |
| Influenza Infection | R-HSA-168255 | 177 | 10337 |
| Interferon gamma signaling | R-HSA-877300 | 176 | 410 |
| SARS-CoV Infections | R-HSA-9679506 | 176 | 981 |
| Cellular Senescence | R-HSA-2559583 | 176 | 3482 |
| Selenoamino acid metabolism | R-HSA-2408522 | 176 | 5596 |
| SLC transporter disorders | R-HSA-5619102 | 175 | 698 |
| Regulation of expression of SLITs and ROBOs | R-HSA-9010553 | 174 | 7238 |
| Separation of Sister Chromatids | R-HSA-2467813 | 174 | 7824 |
| Regulation of TP53 Activity | R-HSA-5633007 | 173 | 1657 |
| ER-Phagosome pathway | R-HSA-1236974 | 173 | 1762 |
| HIV Life Cycle | R-HSA-162587 | 173 | 6044 |
| FCERI mediated NF-kB activation | R-HSA-2871837 | 172 | 3428 |
| G alpha (s) signalling events | R-HSA-418555 | 172 | 3431 |
| HCMV Late Events | R-HSA-9610379 | 170 | 8152 |
| Visual phototransduction | R-HSA-2187338 | 169 | 1740 |
| Protein localization | R-HSA-9609507 | 168 | 1621 |
| Platelet activation, signaling and aggregation | R-HSA-76002 | 168 | 1732 |
| Arachidonic acid metabolism | R-HSA-2142753 | 165 | 1148 |
| Cellular response to starvation | R-HSA-9711097 | 165 | 6951 |
| CDC42 GTPase cycle | R-HSA-9013148 | 163 | 624 |
| Transport of inorganic cations/anions and amino acids/oligopeptides | R-HSA-425393 | 163 | 2579 |
| DNA Double-Strand Break Repair | R-HSA-5693532 | 163 | 4712 |
| Transport of bile salts and organic acids, metal ions and amine compounds | R-HSA-425366 | 162 | 1020 |
| Beta-catenin independent WNT signaling | R-HSA-3858494 | 160 | 2113 |
| Biosynthesis of the N-glycan precursor (dolichol lipid-linked oligosaccharide, LLO) and transfer to a nascent protein | R-HSA-446193 | 159 | 746 |
| Biosynthesis of specialized proresolving mediators (SPMs) | R-HSA-9018678 | 159 | 1255 |
| Clathrin-mediated endocytosis | R-HSA-8856828 | 159 | 10458 |
| Sphingolipid metabolism | R-HSA-428157 | 158 | 845 |
| ADORA2B mediated anti-inflammatory cytokines production | R-HSA-9660821 | 158 | 5364 |
| Regulation of actin dynamics for phagocytic cup formation | R-HSA-2029482 | 158 | 5451 |
| Late Phase of HIV Life Cycle | R-HSA-162599 | 158 | 5756 |
| FCGR3A-mediated phagocytosis | R-HSA-9664422 | 157 | 5999 |
| Complement cascade | R-HSA-166658 | 155 | 3541 |
| Factors involved in megakaryocyte development and platelet production | R-HSA-983231 | 154 | 303 |
| Death Receptor Signalling | R-HSA-73887 | 154 | 1146 |
| G2/M Checkpoints | R-HSA-69481 | 154 | 3062 |
| RHOA GTPase cycle | R-HSA-8980692 | 153 | 581 |
| Mitotic G1 phase and G1/S transition | R-HSA-453279 | 153 | 2916 |
| Influenza Viral RNA Transcription and Replication | R-HSA-168273 | 153 | 8900 |
| Respiratory electron transport, ATP synthesis by chemiosmotic coupling, and heat production by uncoupling proteins. | R-HSA-163200 | 152 | 3573 |
| Interleukin-1 family signaling | R-HSA-446652 | 150 | 2074 |
| Cytoprotection by HMOX1 | R-HSA-9707564 | 150 | 2094 |
| PTEN Regulation | R-HSA-6807070 | 150 | 2271 |
| Toll Like Receptor 4 (TLR4) Cascade | R-HSA-166016 | 148 | 1742 |
| Signaling by Hedgehog | R-HSA-5358351 | 148 | 3052 |
| TCR signaling | R-HSA-202403 | 147 | 2764 |
| Signaling by NTRKs | R-HSA-166520 | 146 | 1093 |
| Autophagy | R-HSA-9612973 | 146 | 2364 |
| ER to Golgi Anterograde Transport | R-HSA-199977 | 145 | 5147 |
| Integration of energy metabolism | R-HSA-163685 | 144 | 1286 |
| Host Interactions of HIV factors | R-HSA-162909 | 144 | 3078 |
| Interferon alpha/beta signaling | R-HSA-909733 | 143 | 1028 |
| DNA Replication | R-HSA-69306 | 141 | 2591 |
| FCGR3A-mediated IL10 synthesis | R-HSA-9664323 | 140 | 4228 |
| Keratinization | R-HSA-6805567 | 138 | 1528 |
| Regulation of Complement cascade | R-HSA-977606 | 138 | 2506 |
| Chromosome Maintenance | R-HSA-73886 | 137 | 3018 |
| Regulation of lipid metabolism by PPARalpha | R-HSA-400206 | 135 | 2252 |
| G1/S Transition | R-HSA-69206 | 135 | 2493 |
| Cardiac conduction | R-HSA-5576891 | 135 | 3276 |
| tRNA processing | R-HSA-72306 | 134 | 949 |
| Glucose metabolism | R-HSA-70326 | 134 | 1475 |
| Epigenetic regulation of gene expression | R-HSA-212165 | 134 | 4483 |
| Nucleobase catabolism | R-HSA-8956319 | 133 | 1048 |
| Transcriptional regulation by RUNX2 | R-HSA-8878166 | 133 | 2115 |
| PPARA activates gene expression | R-HSA-1989781 | 133 | 2236 |
| Signaling by VEGF | R-HSA-194138 | 132 | 1245 |
| O-linked glycosylation | R-HSA-5173105 | 132 | 1306 |
| Synthesis of DNA | R-HSA-69239 | 132 | 2165 |
| Synthesis of substrates in N-glycan biosythesis | R-HSA-446219 | 131 | 578 |
| Macroautophagy | R-HSA-1632852 | 130 | 1658 |
| Homology Directed Repair | R-HSA-5693538 | 130 | 3509 |
| Rab regulation of trafficking | R-HSA-9007101 | 129 | 1089 |
| Golgi-to-ER retrograde transport | R-HSA-8856688 | 129 | 2691 |
| Estrogen-dependent gene expression | R-HSA-9018519 | 128 | 1929 |
| Mitotic Prophase | R-HSA-68875 | 128 | 2367 |
| RHO GTPases Activate Formins | R-HSA-5663220 | 128 | 5505 |
| Regulation of IGF Activity by IGFBP | R-HSA-381426 | 127 | 815 |
| Signaling by NTRK1 (TRKA) | R-HSA-187037 | 126 | 828 |
| MHC class II antigen presentation | R-HSA-2132295 | 126 | 2786 |
| FCERI mediated Ca+2 mobilization | R-HSA-2871809 | 126 | 3696 |
| Negative regulation of the PI3K/AKT network | R-HSA-199418 | 125 | 1793 |
| Cap-dependent Translation Initiation | R-HSA-72737 | 125 | 5797 |
| Eukaryotic Translation Initiation | R-HSA-72613 | 125 | 5823 |
| Downstream TCR signaling | R-HSA-202424 | 124 | 2054 |
| HDR through Homologous Recombination (HRR) or Single Strand Annealing (SSA) | R-HSA-5693567 | 124 | 3416 |
| VEGFA-VEGFR2 Pathway | R-HSA-4420097 | 123 | 1235 |
| Peptide hormone metabolism | R-HSA-2980736 | 122 | 383 |
| Reproduction | R-HSA-1474165 | 122 | 2220 |
| Bile acid and bile salt metabolism | R-HSA-194068 | 121 | 1265 |
| ABC-family proteins mediated transport | R-HSA-382556 | 121 | 1866 |
| FCERI mediated MAPK activation | R-HSA-2871796 | 120 | 3083 |
| Initial triggering of complement | R-HSA-166663 | 119 | 3231 |
| Nucleotide Excision Repair | R-HSA-5696398 | 119 | 4012 |
| Nonsense-Mediated Decay (NMD) | R-HSA-927802 | 119 | 10895 |
| Toll Like Receptor 2 (TLR2) Cascade | R-HSA-181438 | 118 | 1170 |
| Platelet homeostasis | R-HSA-418346 | 117 | 924 |
| PI5P, PP2A and IER3 Regulate PI3K/AKT Signaling | R-HSA-6811558 | 117 | 1584 |
| Respiratory electron transport | R-HSA-611105 | 117 | 2606 |
| GTP hydrolysis and joining of the 60S ribosomal subunit | R-HSA-72706 | 116 | 5482 |
| Post-translational modification: synthesis of GPI-anchored proteins | R-HSA-163125 | 115 | 467 |
| Cargo recognition for clathrin-mediated endocytosis | R-HSA-8856825 | 115 | 1327 |
| Stimuli-sensing channels | R-HSA-2672351 | 114 | 743 |
| CLEC7A (Dectin-1) signaling | R-HSA-5607764 | 114 | 1915 |
| Interleukin-4 and Interleukin-13 signaling | R-HSA-6785807 | 113 | 235 |
| Degradation of the extracellular matrix | R-HSA-1474228 | 113 | 322 |
| TP53 Regulates Metabolic Genes | R-HSA-5628897 | 113 | 893 |
| SRP-dependent cotranslational protein targeting to membrane | R-HSA-1799339 | 113 | 6181 |
| Resolution of Sister Chromatid Cohesion | R-HSA-2500257 | 113 | 6374 |
| Opioid Signalling | R-HSA-111885 | 112 | 1215 |
| PI Metabolism | R-HSA-1483255 | 112 | 1549 |
| PI3K/AKT Signaling in Cancer | R-HSA-2219528 | 111 | 599 |
| Synthesis of bile acids and bile salts | R-HSA-192105 | 111 | 983 |
| HATs acetylate histones | R-HSA-3214847 | 110 | 1034 |
| Telomere Maintenance | R-HSA-157579 | 110 | 1928 |
| Post-translational protein phosphorylation | R-HSA-8957275 | 109 | 744 |
| L1CAM interactions | R-HSA-373760 | 109 | 772 |
| Signaling by TGFB family members | R-HSA-9006936 | 109 | 977 |
| Gene Silencing by RNA | R-HSA-211000 | 109 | 1567 |
| Interleukin-1 signaling | R-HSA-9020702 | 109 | 1905 |
| Mitochondrial biogenesis | R-HSA-1592230 | 108 | 571 |
| p75 NTR receptor-mediated signalling | R-HSA-193704 | 107 | 554 |
| TRIF(TICAM1)-mediated TLR4 signaling | R-HSA-937061 | 107 | 1167 |
| Potassium Channels | R-HSA-1296071 | 107 | 1348 |
| Hedgehog 'off' state | R-HSA-5610787 | 107 | 1778 |
| Toll Like Receptor 9 (TLR9) Cascade | R-HSA-168138 | 106 | 852 |
| Regulation of insulin secretion | R-HSA-422356 | 106 | 1016 |
| Mitochondrial Fatty Acid Beta-Oxidation | R-HSA-77289 | 105 | 780 |
| Glycolysis | R-HSA-70171 | 104 | 1073 |
| Collagen formation | R-HSA-1474290 | 104 | 2835 |
| Response of EIF2AK4 (GCN2) to amino acid deficiency | R-HSA-9633012 | 104 | 4746 |
| Toll Like Receptor 7/8 (TLR7/8) Cascade | R-HSA-168181 | 103 | 850 |
| Transcriptional regulation by RUNX3 | R-HSA-8878159 | 103 | 1348 |
| RUNX1 regulates transcription of genes involved in differentiation of HSCs | R-HSA-8939236 | 103 | 1972 |
| TNFR2 non-canonical NF-kB pathway | R-HSA-5668541 | 103 | 2678 |
| Antigen activates B Cell Receptor (BCR) leading to generation of second messengers | R-HSA-983695 | 103 | 3772 |
| MyD88 dependent cascade initiated on endosome | R-HSA-975155 | 102 | 844 |
| Toll Like Receptor 3 (TLR3) Cascade | R-HSA-168164 | 102 | 871 |
| Oxidative Stress Induced Senescence | R-HSA-2559580 | 102 | 1921 |
| TRAF6 mediated induction of NFkB and MAP kinases upon TLR7/8 or 9 activation | R-HSA-975138 | 101 | 794 |
| Extra-nuclear estrogen signaling | R-HSA-9009391 | 101 | 852 |
| EPH-Ephrin signaling | R-HSA-2682334 | 101 | 998 |
| Antigen Presentation: Folding, assembly and peptide loading of class I MHC | R-HSA-983170 | 101 | 2407 |
| RAC3 GTPase cycle | R-HSA-9013423 | 100 | 347 |
| SARS-CoV-2 Infection | R-HSA-9694516 | 100 | 512 |
| Cellular response to heat stress | R-HSA-3371556 | 100 | 2168 |
| Neurotransmitter release cycle | R-HSA-112310 | 99 | 1359 |
| PCP/CE pathway | R-HSA-4086400 | 99 | 1426 |
| Anchoring of the basal body to the plasma membrane | R-HSA-5620912 | 99 | 4902 |
| Mitochondrial translation | R-HSA-5368287 | 99 | 7044 |
| UCH proteinases | R-HSA-5689603 | 98 | 1551 |
| ABC transporter disorders | R-HSA-5619084 | 98 | 1557 |
| Eukaryotic Translation Elongation | R-HSA-156842 | 98 | 4642 |
| Pyruvate metabolism and Citric Acid (TCA) cycle | R-HSA-71406 | 97 | 1030 |
| Costimulation by the CD28 family | R-HSA-388841 | 97 | 1392 |
| Signaling by FGFR | R-HSA-190236 | 97 | 2360 |
| Regulation of TP53 Activity through Phosphorylation | R-HSA-6804756 | 95 | 975 |
| Unfolded Protein Response (UPR) | R-HSA-381119 | 94 | 216 |
| Sulfur amino acid metabolism | R-HSA-1614635 | 94 | 441 |
| Toll Like Receptor 5 (TLR5) Cascade | R-HSA-168176 | 94 | 767 |
| MyD88 cascade initiated on plasma membrane | R-HSA-975871 | 94 | 767 |
| MAPK6/MAPK4 signaling | R-HSA-5687128 | 94 | 1427 |
| Transcriptional regulation of white adipocyte differentiation | R-HSA-381340 | 94 | 1696 |
| Switching of origins to a post-replicative state | R-HSA-69052 | 93 | 1765 |
| Peptide chain elongation | R-HSA-156902 | 93 | 4622 |
| Mitochondrial translation initiation | R-HSA-5368286 | 93 | 6037 |
| RAC2 GTPase cycle | R-HSA-9013404 | 92 | 321 |
| Lipoprotein metabolism | R-HSA-174824 | 92 | 573 |
| Global Genome Nucleotide Excision Repair (GG-NER) | R-HSA-5696399 | 92 | 1891 |
| Meiosis | R-HSA-1500620 | 92 | 2131 |
| Regulation of PLK1 Activity at G2/M Transition | R-HSA-2565942 | 92 | 2917 |
| Activation of HOX genes during differentiation | R-HSA-5619507 | 92 | 4792 |
| APC/C-mediated degradation of cell cycle proteins | R-HSA-174143 | 92 | 5542 |
| Activation of NMDA receptors and postsynaptic events | R-HSA-442755 | 91 | 1303 |
| Mitochondrial translation elongation | R-HSA-5389840 | 91 | 4608 |
| Mitochondrial translation termination | R-HSA-5419276 | 91 | 4697 |
| Peroxisomal lipid metabolism | R-HSA-390918 | 90 | 377 |
| Inositol phosphate metabolism | R-HSA-1483249 | 90 | 879 |
| Signaling by Insulin receptor | R-HSA-74752 | 90 | 942 |
| Pre-NOTCH Expression and Processing | R-HSA-1912422 | 90 | 1155 |
| Antiviral mechanism by IFN-stimulated genes | R-HSA-1169410 | 90 | 1240 |
| Regulation of mRNA stability by proteins that bind AU-rich elements | R-HSA-450531 | 90 | 1265 |
| Downstream signaling events of B Cell Receptor (BCR) | R-HSA-1168372 | 90 | 1614 |
| Processing of DNA double-strand break ends | R-HSA-5693607 | 90 | 1890 |
| Biosynthesis of DHA-derived SPMs | R-HSA-9018677 | 89 | 487 |
| Metabolism of fat-soluble vitamins | R-HSA-6806667 | 89 | 1164 |
| Cyclin A:Cdk2-associated events at S phase entry | R-HSA-69656 | 89 | 1851 |
| Oncogenic MAPK signaling | R-HSA-6802957 | 89 | 3389 |
| SARS-CoV-1 Infection | R-HSA-9678108 | 88 | 419 |
| DNA Replication Pre-Initiation | R-HSA-69002 | 88 | 1751 |
| COPI-dependent Golgi-to-ER retrograde traffic | R-HSA-6811434 | 88 | 1842 |
| Heparan sulfate/heparin (HS-GAG) metabolism | R-HSA-1638091 | 87 | 769 |
| RNA Polymerase I Transcription | R-HSA-73864 | 87 | 1296 |
| Cyclin E associated events during G1/S transition | R-HSA-69202 | 87 | 1843 |
| Glycosphingolipid metabolism | R-HSA-1660662 | 86 | 381 |
| DDX58/IFIH1-mediated induction of interferon-alpha/beta | R-HSA-168928 | 86 | 602 |
| Nuclear Envelope (NE) Reassembly | R-HSA-2995410 | 86 | 958 |
| RNA Polymerase I Promoter Clearance | R-HSA-73854 | 86 | 1237 |
| Hedgehog 'on' state | R-HSA-5632684 | 86 | 2023 |
| Transport of Mature Transcript to Cytoplasm | R-HSA-72202 | 86 | 4824 |
| G alpha (12/13) signalling events | R-HSA-416482 | 85 | 783 |
| Senescence-Associated Secretory Phenotype (SASP) | R-HSA-2559582 | 85 | 1096 |
| Signaling by NOTCH4 | R-HSA-9013694 | 85 | 1253 |
| Degradation of beta-catenin by the destruction complex | R-HSA-195253 | 85 | 2428 |
| Transcription-Coupled Nucleotide Excision Repair (TC-NER) | R-HSA-6781827 | 85 | 3126 |
| Nicotinate metabolism | R-HSA-196807 | 84 | 394 |
| Formation of the cornified envelope | R-HSA-6809371 | 84 | 800 |
| Sensory processing of sound | R-HSA-9659379 | 84 | 2065 |
| Regulation of APC/C activators between G1/S and early anaphase | R-HSA-176408 | 83 | 1896 |
| Negative epigenetic regulation of rRNA expression | R-HSA-5250941 | 83 | 2713 |
| Centrosome maturation | R-HSA-380287 | 83 | 3566 |
| Protein ubiquitination | R-HSA-8852135 | 82 | 1300 |
| Circadian Clock | R-HSA-400253 | 81 | 452 |
| Interconversion of nucleotide di- and triphosphates | R-HSA-499943 | 81 | 1119 |
| G2/M DNA damage checkpoint | R-HSA-69473 | 81 | 1473 |
| Signaling by FGFR2 | R-HSA-5654738 | 81 | 1820 |
| Cellular response to hypoxia | R-HSA-1234174 | 81 | 2418 |
| Recruitment of mitotic centrosome proteins and complexes | R-HSA-380270 | 81 | 3357 |
| Transcription of the HIV genome | R-HSA-167172 | 81 | 3999 |
| Cell death signalling via NRAGE, NRIF and NADE | R-HSA-204998 | 80 | 385 |
| Sphingolipid de novo biosynthesis | R-HSA-1660661 | 80 | 432 |
| ROS and RNS production in phagocytes | R-HSA-1222556 | 80 | 748 |
| Signaling by TGF-beta Receptor Complex | R-HSA-170834 | 80 | 757 |
| Metabolism of polyamines | R-HSA-351202 | 80 | 1284 |
| Regulation of RUNX2 expression and activity | R-HSA-8939902 | 80 | 1951 |
| RNA polymerase II transcribes snRNA genes | R-HSA-6807505 | 80 | 4481 |
| Fatty acyl-CoA biosynthesis | R-HSA-75105 | 79 | 459 |
| Iron uptake and transport | R-HSA-917937 | 79 | 713 |
| Retinoid metabolism and transport | R-HSA-975634 | 79 | 1144 |
| ISG15 antiviral mechanism | R-HSA-1169408 | 79 | 1208 |
| Positive epigenetic regulation of rRNA expression | R-HSA-5250913 | 79 | 2275 |
| Diseases associated with O-glycosylation of proteins | R-HSA-3906995 | 78 | 132 |
| RHOG GTPase cycle | R-HSA-9013408 | 78 | 221 |
| RHOC GTPase cycle | R-HSA-9013106 | 78 | 260 |
| FOXO-mediated transcription | R-HSA-9614085 | 78 | 264 |
| Diseases of programmed cell death | R-HSA-9645723 | 78 | 754 |
| RHO GTPases activate PKNs | R-HSA-5625740 | 78 | 1307 |
| Regulation of HSF1-mediated heat shock response | R-HSA-3371453 | 78 | 2041 |
| Interleukin-17 signaling | R-HSA-448424 | 77 | 496 |
| Cytosolic sensors of pathogen-associated DNA | R-HSA-1834949 | 77 | 585 |
| Signaling by NOTCH1 | R-HSA-1980143 | 77 | 591 |
| SUMOylation of DNA damage response and repair proteins | R-HSA-3108214 | 77 | 841 |
| Transcriptional regulation by small RNAs | R-HSA-5578749 | 77 | 1432 |
| NoRC negatively regulates rRNA expression | R-HSA-427413 | 77 | 2264 |
| COPII-mediated vesicle transport | R-HSA-204005 | 77 | 2317 |
| Activation of APC/C and APC/C:Cdc20 mediated degradation of mitotic proteins | R-HSA-176814 | 77 | 3312 |
| Transport of Mature mRNA derived from an Intron-Containing Transcript | R-HSA-159236 | 77 | 4454 |
| Synthesis of bile acids and bile salts via 7alpha-hydroxycholesterol | R-HSA-193368 | 76 | 492 |
| Collagen biosynthesis and modifying enzymes | R-HSA-1650814 | 76 | 2725 |
| APC/C:Cdc20 mediated degradation of mitotic proteins | R-HSA-176409 | 76 | 3261 |
| Signaling by MET | R-HSA-6806834 | 75 | 594 |
| Ca2+ pathway | R-HSA-4086398 | 75 | 700 |
| CDK-mediated phosphorylation and removal of Cdc6 | R-HSA-69017 | 75 | 1511 |
| RHOB GTPase cycle | R-HSA-9013026 | 74 | 267 |
| Gamma carboxylation, hypusine formation and arylsulfatase activation | R-HSA-163841 | 74 | 309 |
| Post NMDA receptor activation events | R-HSA-438064 | 74 | 921 |
| Regulation of PTEN stability and activity | R-HSA-8948751 | 74 | 1326 |
| APC:Cdc20 mediated degradation of cell cycle proteins prior to satisfation of the cell cycle checkpoint | R-HSA-179419 | 74 | 2874 |
| APC/C:Cdh1 mediated degradation of Cdc20 and other APC/C:Cdh1 targeted proteins in late mitosis/early G1 | R-HSA-174178 | 74 | 4046 |
| Regulated Necrosis | R-HSA-5218859 | 73 | 300 |
| Porphyrin metabolism | R-HSA-189445 | 73 | 309 |
| Chondroitin sulfate/dermatan sulfate metabolism | R-HSA-1793185 | 73 | 534 |
| Xenobiotics | R-HSA-211981 | 73 | 1076 |
| O-linked glycosylation of mucins | R-HSA-913709 | 73 | 1090 |
| Orc1 removal from chromatin | R-HSA-68949 | 73 | 1438 |
| Sensory processing of sound by inner hair cells of the cochlea | R-HSA-9662360 | 73 | 1745 |
| Cdc20:Phospho-APC/C mediated degradation of Cyclin A | R-HSA-174184 | 73 | 2820 |
| Glyoxylate metabolism and glycine degradation | R-HSA-389661 | 72 | 274 |
| G-protein mediated events | R-HSA-112040 | 72 | 649 |
| Signaling by FGFR in disease | R-HSA-1226099 | 72 | 1131 |
| Activation of NF-kappaB in B cells | R-HSA-1169091 | 72 | 1470 |
| Hedgehog ligand biogenesis | R-HSA-5358346 | 72 | 1563 |
| HDR through Homologous Recombination (HRR) | R-HSA-5685942 | 72 | 1953 |
| Oxygen-dependent proline hydroxylation of Hypoxia-inducible Factor Alpha | R-HSA-1234176 | 72 | 2370 |
| Cholesterol biosynthesis | R-HSA-191273 | 71 | 362 |
| Cytosolic sulfonation of small molecules | R-HSA-156584 | 71 | 373 |
| Signaling by Retinoic Acid | R-HSA-5362517 | 71 | 531 |
| Ion transport by P-type ATPases | R-HSA-936837 | 71 | 699 |
| Loss of Nlp from mitotic centrosomes | R-HSA-380259 | 71 | 2555 |
| Semaphorin interactions | R-HSA-373755 | 70 | 706 |
| Metabolism of steroid hormones | R-HSA-196071 | 70 | 794 |
| Regulation of RAS by GAPs | R-HSA-5658442 | 70 | 1385 |
| G1/S DNA Damage Checkpoints | R-HSA-69615 | 70 | 1388 |
| DNA Double Strand Break Response | R-HSA-5693606 | 70 | 1879 |
| Signaling by BRAF and RAF fusions | R-HSA-6802952 | 70 | 2154 |
| MAP kinase activation | R-HSA-450294 | 69 | 484 |
| Mitochondrial protein import | R-HSA-1268020 | 69 | 680 |
| Synthesis of PIPs at the plasma membrane | R-HSA-1660499 | 69 | 857 |
| Transcriptional activation of mitochondrial biogenesis | R-HSA-2151201 | 68 | 291 |
| Nucleotide salvage | R-HSA-8956321 | 68 | 332 |
| Amyloid fiber formation | R-HSA-977225 | 68 | 453 |
| tRNA Aminoacylation | R-HSA-379724 | 68 | 459 |
| Regulation of PTEN gene transcription | R-HSA-8943724 | 68 | 462 |
| Extension of Telomeres | R-HSA-180786 | 68 | 1266 |
| p53-Dependent G1/S DNA damage checkpoint | R-HSA-69580 | 68 | 1329 |
| Assembly of the pre-replicative complex | R-HSA-68867 | 68 | 1332 |
| Regulation of HMOX1 expression and activity | R-HSA-9707587 | 68 | 1366 |
| rRNA modification in the nucleus and cytosol | R-HSA-6790901 | 68 | 1480 |
| RUNX1 regulates genes involved in megakaryocyte differentiation and platelet function | R-HSA-8936459 | 68 | 2116 |
| APC/C:Cdc20 mediated degradation of Securin | R-HSA-174154 | 68 | 2456 |
| Glutathione conjugation | R-HSA-156590 | 67 | 350 |
| PLC beta mediated events | R-HSA-112043 | 67 | 426 |
| Signaling by ERBB4 | R-HSA-1236394 | 67 | 437 |
| Selective autophagy | R-HSA-9663891 | 67 | 469 |
| Amino acid transport across the plasma membrane | R-HSA-352230 | 67 | 719 |
| GABA receptor activation | R-HSA-977443 | 67 | 782 |
| TP53 Regulates Transcription of DNA Repair Genes | R-HSA-6796648 | 67 | 1666 |
| Recruitment and ATM-mediated phosphorylation of repair and signaling proteins at DNA double strand breaks | R-HSA-5693565 | 67 | 1853 |
| RNA Polymerase II Transcription Termination | R-HSA-73856 | 67 | 1918 |
| Triglyceride metabolism | R-HSA-8979227 | 66 | 247 |
| Nucleotide-binding domain, leucine rich repeat containing receptor (NLR) signaling pathways | R-HSA-168643 | 66 | 274 |
| Metabolism of cofactors | R-HSA-8978934 | 66 | 307 |
| Gluconeogenesis | R-HSA-70263 | 66 | 341 |
| Peroxisomal protein import | R-HSA-9033241 | 66 | 581 |
| Pre-NOTCH Transcription and Translation | R-HSA-1912408 | 66 | 1008 |
| Asymmetric localization of PCP proteins | R-HSA-4608870 | 66 | 1127 |
| Defective CFTR causes cystic fibrosis | R-HSA-5678895 | 66 | 1502 |
| RNA Polymerase II Transcription Elongation | R-HSA-75955 | 66 | 2154 |
| Gap-filling DNA repair synthesis and ligation in TC-NER | R-HSA-6782210 | 66 | 2234 |
| Nuclear Events (kinase and transcription factor activation) | R-HSA-198725 | 65 | 230 |
| Regulation of cholesterol biosynthesis by SREBP (SREBF) | R-HSA-1655829 | 65 | 380 |
| Synthesis of PA | R-HSA-1483166 | 65 | 417 |
| Deadenylation-dependent mRNA decay | R-HSA-429914 | 65 | 623 |
| Insulin receptor signalling cascade | R-HSA-74751 | 65 | 631 |
| DNA Damage/Telomere Stress Induced Senescence | R-HSA-2559586 | 65 | 704 |
| Heme signaling | R-HSA-9707616 | 64 | 315 |
| Signaling by PTK6 | R-HSA-8848021 | 64 | 386 |
| Signaling by Non-Receptor Tyrosine Kinases | R-HSA-9006927 | 64 | 386 |
| Signaling by Type 1 Insulin-like Growth Factor 1 Receptor (IGF1R) | R-HSA-2404192 | 64 | 600 |
| tRNA processing in the nucleus | R-HSA-6784531 | 64 | 768 |
| Formation of the beta-catenin:TCF transactivating complex | R-HSA-201722 | 64 | 1088 |
| Hh mutants abrogate ligand secretion | R-HSA-5387390 | 64 | 1365 |
| Autodegradation of Cdh1 by Cdh1:APC/C | R-HSA-174084 | 64 | 2103 |
| RHOQ GTPase cycle | R-HSA-9013406 | 63 | 179 |
| Transcriptional Regulation by MECP2 | R-HSA-8986944 | 63 | 206 |
| NRAGE signals death through JNK | R-HSA-193648 | 63 | 295 |
| Signaling by PDGF | R-HSA-186797 | 63 | 304 |
| NCAM signaling for neurite out-growth | R-HSA-375165 | 63 | 350 |
| Detoxification of Reactive Oxygen Species | R-HSA-3299685 | 63 | 429 |
| Signaling by ERBB2 | R-HSA-1227986 | 63 | 768 |
| Formation of RNA Pol II elongation complex | R-HSA-112382 | 63 | 1932 |
| HDACs deacetylate histones | R-HSA-3214815 | 63 | 2146 |
| Nucleobase biosynthesis | R-HSA-8956320 | 62 | 378 |
| Ion homeostasis | R-HSA-5578775 | 62 | 1047 |
| Degradation of GLI2 by the proteasome | R-HSA-5610783 | 62 | 1190 |
| GLI3 is processed to GLI3R by the proteasome | R-HSA-5610785 | 62 | 1234 |
| The role of GTSE1 in G2/M progression after G2 checkpoint | R-HSA-8852276 | 62 | 1306 |
| Degradation of GLI1 by the proteasome | R-HSA-5610780 | 62 | 1333 |
| Meiotic synapsis | R-HSA-1221632 | 62 | 1378 |
| Dectin-1 mediated noncanonical NF-kB signaling | R-HSA-5607761 | 62 | 1402 |
| B-WICH complex positively regulates rRNA expression | R-HSA-5250924 | 62 | 1649 |
| SCF(Skp2)-mediated degradation of p27/p21 | R-HSA-187577 | 62 | 1715 |
| Signaling by NOTCH1 PEST Domain Mutants in Cancer | R-HSA-2644602 | 61 | 405 |
| Signaling by NOTCH1 HD+PEST Domain Mutants in Cancer | R-HSA-2894858 | 61 | 432 |
| Signaling by NOTCH1 in Cancer | R-HSA-2644603 | 61 | 456 |
| Hh mutants are degraded by ERAD | R-HSA-5362768 | 61 | 1364 |
| NIK-->noncanonical NF-kB signaling | R-HSA-5676590 | 61 | 1401 |
| Sensory processing of sound by outer hair cells of the cochlea | R-HSA-9662361 | 61 | 1579 |
| Amino acids regulate mTORC1 | R-HSA-9639288 | 61 | 2205 |
| Intrinsic Pathway for Apoptosis | R-HSA-109606 | 60 | 214 |
| Sialic acid metabolism | R-HSA-4085001 | 60 | 317 |
| Transcriptional regulation of granulopoiesis | R-HSA-9616222 | 60 | 557 |
| Nuclear Envelope Breakdown | R-HSA-2980766 | 60 | 856 |
| ROS sensing by NFE2L2 | R-HSA-8932339 | 60 | 1295 |
| Assembly of collagen fibrils and other multimeric structures | R-HSA-2022090 | 59 | 133 |
| RHOJ GTPase cycle | R-HSA-9013409 | 59 | 169 |
| Nicotinamide salvaging | R-HSA-197264 | 59 | 198 |
| The phototransduction cascade | R-HSA-2514856 | 59 | 355 |
| Generation of second messenger molecules | R-HSA-202433 | 59 | 770 |
| E3 ubiquitin ligases ubiquitinate target proteins | R-HSA-8866654 | 59 | 1168 |
| Signaling by EGFR | R-HSA-177929 | 59 | 1178 |
| CDT1 association with the CDC6:ORC:origin complex | R-HSA-68827 | 59 | 1260 |
| Stabilization of p53 | R-HSA-69541 | 59 | 1299 |
| Interleukin-12 family signaling | R-HSA-447115 | 58 | 266 |
| Netrin-1 signaling | R-HSA-373752 | 58 | 328 |
| Branched-chain amino acid catabolism | R-HSA-70895 | 58 | 455 |
| Translocation of SLC2A4 (GLUT4) to the plasma membrane | R-HSA-1445148 | 58 | 485 |
| SUMOylation of chromatin organization proteins | R-HSA-4551638 | 58 | 716 |
| Meiotic recombination | R-HSA-912446 | 58 | 982 |
| Signaling by FGFR1 | R-HSA-5654736 | 58 | 1119 |
| mRNA 3'-end processing | R-HSA-72187 | 58 | 1913 |
| Synthesis of Prostaglandins (PG) and Thromboxanes (TX) | R-HSA-2162123 | 57 | 214 |
| The canonical retinoid cycle in rods (twilight vision) | R-HSA-2453902 | 57 | 254 |
| N-glycan trimming in the ER and Calnexin/Calreticulin cycle | R-HSA-532668 | 57 | 504 |
| DNA Damage Bypass | R-HSA-73893 | 57 | 1117 |
| Regulation of RUNX3 expression and activity | R-HSA-8941858 | 57 | 1129 |
| Degradation of AXIN | R-HSA-4641257 | 57 | 1547 |
| Degradation of DVL | R-HSA-4641258 | 57 | 1593 |
| Non-coding RNA Metabolism | R-HSA-194441 | 57 | 1977 |
| Metabolism of amine-derived hormones | R-HSA-209776 | 56 | 277 |
| Glucuronidation | R-HSA-156588 | 56 | 367 |
| Pyruvate metabolism | R-HSA-70268 | 56 | 525 |
| Negative regulation of NOTCH4 signaling | R-HSA-9604323 | 56 | 1113 |
| Formation of TC-NER Pre-Incision Complex | R-HSA-6781823 | 56 | 1315 |
| Diseases associated with glycosaminoglycan metabolism | R-HSA-3560782 | 55 | 170 |
| Synthesis of PC | R-HSA-1483191 | 55 | 206 |
| Cargo trafficking to the periciliary membrane | R-HSA-5620920 | 55 | 731 |
| Retrograde transport at the Trans-Golgi-Network | R-HSA-6811440 | 55 | 850 |
| AUF1 (hnRNP D0) binds and destabilizes mRNA | R-HSA-450408 | 55 | 1059 |
| SCF-beta-TrCP mediated degradation of Emi1 | R-HSA-174113 | 55 | 1508 |
| Vif-mediated degradation of APOBEC3G | R-HSA-180585 | 55 | 1512 |
| FBXL7 down-regulates AURKA during mitotic entry and in early mitosis | R-HSA-8854050 | 55 | 1525 |
| RNA Polymerase II Transcription Initiation And Promoter Clearance | R-HSA-76042 | 55 | 2641 |
| Apoptotic execution phase | R-HSA-75153 | 54 | 80 |
| TP53 Regulates Transcription of Cell Death Genes | R-HSA-5633008 | 54 | 97 |
| Inactivation, recovery and regulation of the phototransduction cascade | R-HSA-2514859 | 54 | 250 |
| Regulation of Apoptosis | R-HSA-169911 | 54 | 1227 |
| p53-Independent DNA Damage Response | R-HSA-69610 | 54 | 1232 |
| Ubiquitin-dependent degradation of Cyclin D | R-HSA-75815 | 54 | 1233 |
| Autodegradation of the E3 ubiquitin ligase COP1 | R-HSA-349425 | 54 | 1234 |
| DAG and IP3 signaling | R-HSA-1489509 | 53 | 321 |
| EPH-ephrin mediated repulsion of cells | R-HSA-3928665 | 53 | 372 |
| RMTs methylate histone arginines | R-HSA-3214858 | 53 | 397 |
| Condensation of Prophase Chromosomes | R-HSA-2299718 | 53 | 1393 |
| Vpu mediated degradation of CD4 | R-HSA-180534 | 53 | 1591 |
| RNA Polymerase II Promoter Escape | R-HSA-73776 | 53 | 2331 |
| IRE1alpha activates chaperones | R-HSA-381070 | 52 | 56 |
| Signaling by NOTCH3 | R-HSA-9012852 | 52 | 287 |
| Synthesis of Leukotrienes (LT) and Eoxins (EX) | R-HSA-2142691 | 52 | 321 |
| Signaling by FGFR2 in disease | R-HSA-5655253 | 52 | 696 |
| Tat-mediated elongation of the HIV-1 transcript | R-HSA-167246 | 52 | 1329 |
| HIV Transcription Elongation | R-HSA-167169 | 52 | 1363 |
| RNA Polymerase II HIV Promoter Escape | R-HSA-167162 | 52 | 2270 |
| TP53 Regulates Transcription of Cell Cycle Genes | R-HSA-6791312 | 51 | 168 |
| Synthesis of very long-chain fatty acyl-CoAs | R-HSA-75876 | 51 | 278 |
| Keratan sulfate/keratin metabolism | R-HSA-1638074 | 51 | 294 |
| G1 Phase | R-HSA-69236 | 51 | 423 |
| LXR-mediated signaling | R-HSA-9024446 | 51 | 443 |
| Carboxyterminal post-translational modifications of tubulin | R-HSA-8955332 | 51 | 451 |
| DAP12 interactions | R-HSA-2172127 | 51 | 508 |
| Telomere C-strand (Lagging Strand) Synthesis | R-HSA-174417 | 51 | 921 |
| Regulation of ornithine decarboxylase (ODC) | R-HSA-350562 | 51 | 1181 |
| Transcriptional activity of SMAD2/SMAD3:SMAD4 heterotrimer | R-HSA-2173793 | 50 | 290 |
| NOTCH1 Intracellular Domain Regulates Transcription | R-HSA-2122947 | 50 | 308 |
| Cytosolic tRNA aminoacylation | R-HSA-379716 | 50 | 309 |
| Citric acid cycle (TCA cycle) | R-HSA-71403 | 50 | 364 |
| Platelet Aggregation (Plug Formation) | R-HSA-76009 | 50 | 487 |
| TNF signaling | R-HSA-75893 | 50 | 568 |
| MTOR signalling | R-HSA-165159 | 50 | 810 |
| Signaling by FGFR3 | R-HSA-5654741 | 50 | 873 |
| Formation of HIV elongation complex in the absence of HIV Tat | R-HSA-167152 | 50 | 1220 |
| Regulation of activated PAK-2p34 by proteasome mediated degradation | R-HSA-211733 | 50 | 1223 |
| Paradoxical activation of RAF signaling by kinase inactive BRAF | R-HSA-6802955 | 50 | 1422 |
| Signaling by moderate kinase activity BRAF mutants | R-HSA-6802946 | 50 | 1426 |
| Signaling by RAS mutants | R-HSA-6802949 | 50 | 1430 |
| Signaling downstream of RAS mutants | R-HSA-9649948 | 50 | 1430 |
| HSP90 chaperone cycle for SHRs | R-HSA-3371497 | 50 | 1448 |
| PKMTs methylate histone lysines | R-HSA-3214841 | 49 | 237 |
| Aflatoxin activation and detoxification | R-HSA-5423646 | 49 | 268 |
| Activation of gene expression by SREBF (SREBP) | R-HSA-2426168 | 49 | 293 |
| Signaling by SCF-KIT | R-HSA-1433557 | 49 | 355 |
| RNA Polymerase I Transcription Initiation | R-HSA-73762 | 49 | 650 |
| GABA B receptor activation | R-HSA-977444 | 49 | 664 |
| Smooth Muscle Contraction | R-HSA-445355 | 49 | 807 |
| Signaling by FGFR4 | R-HSA-5654743 | 49 | 972 |
| Formation of HIV-1 elongation complex containing HIV-1 Tat | R-HSA-167200 | 49 | 1189 |
| XBP1(S) activates chaperone genes | R-HSA-381038 | 48 | 47 |
| Interleukin-12 signaling | R-HSA-9020591 | 48 | 132 |
| Blood group systems biosynthesis | R-HSA-9033658 | 48 | 230 |
| Plasma lipoprotein remodeling | R-HSA-8963899 | 48 | 270 |
| Ca-dependent events | R-HSA-111996 | 48 | 288 |
| Negative regulation of MAPK pathway | R-HSA-5675221 | 48 | 364 |
| Intra-Golgi traffic | R-HSA-6811438 | 48 | 444 |
| Inhibition of DNA recombination at telomere | R-HSA-9670095 | 48 | 617 |
| Translesion synthesis by Y family DNA polymerases bypasses lesions on DNA template | R-HSA-110313 | 48 | 865 |
| Interleukin-10 signaling | R-HSA-6783783 | 47 | 79 |
| Mitochondrial tRNA aminoacylation | R-HSA-379726 | 47 | 150 |
| SUMOylation of RNA binding proteins | R-HSA-4570464 | 47 | 616 |
| Formation of Incision Complex in GG-NER | R-HSA-5696395 | 47 | 783 |
| Activation of rRNA Expression by ERCC6 (CSB) and EHMT2 (G9a) | R-HSA-427389 | 47 | 910 |
| Activated PKN1 stimulates transcription of AR (androgen receptor) regulated genes KLK2 and KLK3 | R-HSA-5625886 | 47 | 1120 |
| Surfactant metabolism | R-HSA-5683826 | 46 | 201 |
| Pentose phosphate pathway | R-HSA-71336 | 46 | 222 |
| Signaling by FGFR1 in disease | R-HSA-5655302 | 46 | 673 |
| RNA Polymerase III Transcription | R-HSA-74158 | 46 | 817 |
| Signaling by RAF1 mutants | R-HSA-9656223 | 46 | 836 |
| SUMOylation of DNA replication proteins | R-HSA-4615885 | 46 | 1047 |
| MAP2K and MAPK activation | R-HSA-5674135 | 46 | 1582 |
| Incretin synthesis, secretion, and inactivation | R-HSA-400508 | 45 | 158 |
| FLT3 Signaling | R-HSA-9607240 | 45 | 221 |
| PD-1 signaling | R-HSA-389948 | 45 | 779 |
| Phosphorylation of CD3 and TCR zeta chains | R-HSA-202427 | 45 | 932 |
| Regulation of beta-cell development | R-HSA-186712 | 44 | 103 |
| RIPK1-mediated regulated necrosis | R-HSA-5213460 | 44 | 176 |
| SUMOylation of transcription cofactors | R-HSA-3899300 | 44 | 210 |
| Deactivation of the beta-catenin transactivating complex | R-HSA-3769402 | 44 | 228 |
| VEGFR2 mediated vascular permeability | R-HSA-5218920 | 44 | 363 |
| COPI-independent Golgi-to-ER retrograde traffic | R-HSA-6811436 | 44 | 849 |
| Ovarian tumor domain proteases | R-HSA-5689896 | 43 | 94 |
| HS-GAG degradation | R-HSA-2024096 | 43 | 173 |
| Glycogen metabolism | R-HSA-8982491 | 43 | 260 |
| Calmodulin induced events | R-HSA-111933 | 43 | 265 |
| CaM pathway | R-HSA-111997 | 43 | 265 |
| Metabolism of folate and pterines | R-HSA-196757 | 43 | 321 |
| Formation of Fibrin Clot (Clotting Cascade) | R-HSA-140877 | 43 | 338 |
| EPHB-mediated forward signaling | R-HSA-3928662 | 43 | 343 |
| Transport of Mature mRNAs Derived from Intronless Transcripts | R-HSA-159234 | 43 | 1046 |
| Synthesis, secretion, and inactivation of Glucagon-like Peptide-1 (GLP-1) | R-HSA-381771 | 42 | 128 |
| Plasma lipoprotein clearance | R-HSA-8964043 | 42 | 208 |
| Regulation of TP53 Expression and Degradation | R-HSA-6806003 | 42 | 249 |
| Acyl chain remodelling of PE | R-HSA-1482839 | 42 | 287 |
| SIRT1 negatively regulates rRNA expression | R-HSA-427359 | 42 | 775 |
| Translocation of ZAP-70 to Immunological synapse | R-HSA-202430 | 42 | 779 |
| Transport of Mature mRNA Derived from an Intronless Transcript | R-HSA-159231 | 42 | 951 |
| NS1 Mediated Effects on Host Pathways | R-HSA-168276 | 42 | 955 |
| Fanconi Anemia Pathway | R-HSA-6783310 | 42 | 1079 |
| Export of Viral Ribonucleoproteins from Nucleus | R-HSA-168274 | 42 | 1093 |
| NGF-stimulated transcription | R-HSA-9031628 | 41 | 100 |
| O-glycosylation of TSR domain-containing proteins | R-HSA-5173214 | 41 | 151 |
| Regulation of TP53 Degradation | R-HSA-6804757 | 41 | 247 |
| Calnexin/calreticulin cycle | R-HSA-901042 | 41 | 305 |
| LXRs regulate gene expression linked to cholesterol transport and efflux | R-HSA-9029569 | 41 | 359 |
| RHO GTPases Activate WASPs and WAVEs | R-HSA-5663213 | 41 | 689 |
| Signaling by high-kinase activity BRAF mutants | R-HSA-6802948 | 41 | 783 |
| NEP/NS2 Interacts with the Cellular Export Machinery | R-HSA-168333 | 41 | 1055 |
| RAS processing | R-HSA-9648002 | 40 | 219 |
| Glucagon signaling in metabolic regulation | R-HSA-163359 | 40 | 226 |
| Regulation of TNFR1 signaling | R-HSA-5357905 | 40 | 252 |
| Acyl chain remodelling of PC | R-HSA-1482788 | 40 | 282 |
| Signalling to ERKs | R-HSA-187687 | 40 | 354 |
| HS-GAG biosynthesis | R-HSA-2022928 | 40 | 371 |
| RAF activation | R-HSA-5673000 | 40 | 481 |
| DNA Damage Recognition in GG-NER | R-HSA-5696394 | 40 | 586 |
| Defective B3GALTL causes Peters-plus syndrome (PpS) | R-HSA-5083635 | 39 | 38 |
| SUMOylation of intracellular receptors | R-HSA-4090294 | 39 | 116 |
| Lewis blood group biosynthesis | R-HSA-9037629 | 39 | 173 |
| Transcriptional Regulation by VENTX | R-HSA-8853884 | 39 | 223 |
| RA biosynthesis pathway | R-HSA-5365859 | 39 | 327 |
| CD28 co-stimulation | R-HSA-389356 | 39 | 339 |
| Regulation of TP53 Activity through Acetylation | R-HSA-6804758 | 39 | 375 |
| Activation of ATR in response to replication stress | R-HSA-176187 | 39 | 391 |
| Glutamate binding, activation of AMPA receptors and synaptic plasticity | R-HSA-399721 | 39 | 520 |
| SUMOylation of ubiquitinylation proteins | R-HSA-3232142 | 39 | 542 |
| HDR through Single Strand Annealing (SSA) | R-HSA-5685938 | 39 | 627 |
| Sealing of the nuclear envelope (NE) by ESCRT-III | R-HSA-9668328 | 39 | 655 |
| Apoptotic cleavage of cellular proteins | R-HSA-111465 | 38 | 44 |
| FGFR2 mutant receptor activation | R-HSA-1839126 | 38 | 261 |
| CREB1 phosphorylation through NMDA receptor-mediated activation of RAS signaling | R-HSA-442742 | 38 | 425 |
| Integrin signaling | R-HSA-354192 | 38 | 460 |
| Negative regulation of FGFR2 signaling | R-HSA-5654727 | 38 | 525 |
| Inwardly rectifying K+ channels | R-HSA-1296065 | 38 | 537 |
| Downstream signaling of activated FGFR1 | R-HSA-5654687 | 38 | 631 |
| Nuclear Pore Complex (NPC) Disassembly | R-HSA-3301854 | 38 | 699 |
| Striated Muscle Contraction | R-HSA-390522 | 38 | 957 |
| Gene and protein expression by JAK-STAT signaling after Interleukin-12 stimulation | R-HSA-8950505 | 37 | 36 |
| Transcriptional regulation by the AP-2 (TFAP2) family of transcription factors | R-HSA-8864260 | 37 | 98 |
| RUNX2 regulates bone development | R-HSA-8941326 | 37 | 110 |
| Keratan sulfate biosynthesis | R-HSA-2022854 | 37 | 239 |
| FLT3 signaling in disease | R-HSA-9682385 | 37 | 258 |
| Recycling of bile acids and salts | R-HSA-159418 | 37 | 338 |
| Negative regulation of FGFR1 signaling | R-HSA-5654726 | 37 | 483 |
| Trafficking of AMPA receptors | R-HSA-399719 | 37 | 498 |
| Downstream signaling of activated FGFR2 | R-HSA-5654696 | 37 | 678 |
| Resolution of D-Loop Structures | R-HSA-5693537 | 37 | 741 |
| EGFR downregulation | R-HSA-182971 | 37 | 833 |
| Synthesis of glycosylphosphatidylinositol (GPI) | R-HSA-162710 | 36 | 130 |
| Negative regulators of RIG-I/MDA5 signaling | R-HSA-936440 | 36 | 136 |
| Transcriptional Regulation by E2F6 | R-HSA-8953750 | 36 | 190 |
| Oncogene Induced Senescence | R-HSA-2559585 | 36 | 212 |
| NOD1/2 Signaling Pathway | R-HSA-168638 | 36 | 229 |
| GABA synthesis, release, reuptake and degradation | R-HSA-888590 | 36 | 295 |
| TGF-beta receptor signaling activates SMADs | R-HSA-2173789 | 36 | 357 |
| Energy dependent regulation of mTOR by LKB1-AMPK | R-HSA-380972 | 36 | 519 |
| Activation of the pre-replicative complex | R-HSA-68962 | 36 | 525 |
| Regulation of Glucokinase by Glucokinase Regulatory Protein | R-HSA-170822 | 36 | 569 |
| Resolution of D-loop Structures through Holliday Junction Intermediates | R-HSA-5693568 | 36 | 712 |
| Transport of the SLBP Dependant Mature mRNA | R-HSA-159230 | 36 | 801 |
| Transport of Ribonucleoproteins into the Host Nucleus | R-HSA-168271 | 36 | 809 |
| Budding and maturation of HIV virion | R-HSA-162588 | 36 | 970 |
| Activation of Matrix Metalloproteinases | R-HSA-1592389 | 35 | 79 |
| Vitamin B5 (pantothenate) metabolism | R-HSA-199220 | 35 | 125 |
| DARPP-32 events | R-HSA-180024 | 35 | 143 |
| Nitric oxide stimulates guanylate cyclase | R-HSA-392154 | 35 | 153 |
| MAPK targets/ Nuclear events mediated by MAP kinases | R-HSA-450282 | 35 | 159 |
| Diseases of mitotic cell cycle | R-HSA-9675126 | 35 | 248 |
| Aberrant regulation of mitotic cell cycle due to RB1 defects | R-HSA-9687139 | 35 | 248 |
| TAK1 activates NFkB by phosphorylation and activation of IKKs complex | R-HSA-445989 | 35 | 263 |
| Endosomal Sorting Complex Required For Transport (ESCRT) | R-HSA-917729 | 35 | 338 |
| Thrombin signalling through proteinase activated receptors (PARs) | R-HSA-456926 | 35 | 340 |
| Platelet calcium homeostasis | R-HSA-418360 | 35 | 431 |
| Negative regulation of FGFR4 signaling | R-HSA-5654733 | 35 | 459 |
| DAP12 signaling | R-HSA-2424491 | 35 | 484 |
| Termination of translesion DNA synthesis | R-HSA-5656169 | 35 | 526 |
| SUMOylation of SUMOylation proteins | R-HSA-4085377 | 35 | 594 |
| Transport of the SLBP independent Mature mRNA | R-HSA-159227 | 35 | 706 |
| FOXO-mediated transcription of oxidative stress, metabolic and neuronal genes | R-HSA-9615017 | 34 | 84 |
| Activation of BH3-only proteins | R-HSA-114452 | 34 | 97 |
| Nuclear signaling by ERBB4 | R-HSA-1251985 | 34 | 98 |
| PERK regulates gene expression | R-HSA-381042 | 34 | 128 |
| SMAD2/SMAD3:SMAD4 heterotrimer regulates transcription | R-HSA-2173796 | 34 | 132 |
| Regulation of MECP2 expression and activity | R-HSA-9022692 | 34 | 140 |
| Signaling by NOTCH2 | R-HSA-1980145 | 34 | 183 |
| Synthesis of bile acids and bile salts via 27-hydroxycholesterol | R-HSA-193807 | 34 | 228 |
| Activation of kainate receptors upon glutamate binding | R-HSA-451326 | 34 | 312 |
| RNA Polymerase I Promoter Opening | R-HSA-73728 | 34 | 322 |
| Recycling pathway of L1 | R-HSA-437239 | 34 | 342 |
| Downstream signaling of activated FGFR4 | R-HSA-5654716 | 34 | 585 |
| Late endosomal microautophagy | R-HSA-9615710 | 34 | 674 |
| BMAL1:CLOCK,NPAS2 activates circadian gene expression | R-HSA-1368108 | 33 | 178 |
| Metal ion SLC transporters | R-HSA-425410 | 33 | 178 |
| Downregulation of ERBB2 signaling | R-HSA-8863795 | 33 | 216 |
| MET promotes cell motility | R-HSA-8875878 | 33 | 235 |
| Formation of the HIV-1 Early Elongation Complex | R-HSA-167158 | 33 | 324 |
| Signaling by ERBB2 in Cancer | R-HSA-1227990 | 33 | 373 |
| Negative regulation of FGFR3 signaling | R-HSA-5654732 | 33 | 415 |
| Disassembly of the destruction complex and recruitment of AXIN to the membrane | R-HSA-4641262 | 33 | 429 |
| Telomere Extension By Telomerase | R-HSA-171319 | 33 | 463 |
| Processive synthesis on the C-strand of the telomere | R-HSA-174414 | 33 | 468 |
| Signaling by CSF3 (G-CSF) | R-HSA-9674555 | 33 | 587 |
| Pyroptosis | R-HSA-5620971 | 32 | 124 |
| Cyclin A/B1/B2 associated events during G2/M transition | R-HSA-69273 | 32 | 195 |
| Synthesis of PIPs at the Golgi membrane | R-HSA-1660514 | 32 | 204 |
| ER Quality Control Compartment (ERQC) | R-HSA-901032 | 32 | 251 |
| Myogenesis | R-HSA-525793 | 32 | 257 |
| Activated NOTCH1 Transmits Signal to the Nucleus | R-HSA-2122948 | 32 | 286 |
| Metalloprotease DUBs | R-HSA-5689901 | 32 | 326 |
| Adrenaline,noradrenaline inhibits insulin secretion | R-HSA-400042 | 32 | 355 |
| Glutamate Neurotransmitter Release Cycle | R-HSA-210500 | 32 | 364 |
| Signaling by ERBB2 KD Mutants | R-HSA-9664565 | 32 | 370 |
| Defective TPR may confer susceptibility towards thyroid papillary carcinoma (TPC) | R-HSA-5619107 | 32 | 495 |
| Downstream signaling of activated FGFR3 | R-HSA-5654708 | 32 | 523 |
| Collagen degradation | R-HSA-1442490 | 31 | 50 |
| Mitophagy | R-HSA-5205647 | 31 | 129 |
| Caspase activation via extrinsic apoptotic signalling pathway | R-HSA-5357769 | 31 | 152 |
| HDMs demethylate histones | R-HSA-3214842 | 31 | 177 |
| Signaling by EGFR in Cancer | R-HSA-1643713 | 31 | 212 |
| Acyl chain remodelling of PS | R-HSA-1482801 | 31 | 219 |
| Synthesis of PIPs at the early endosome membrane | R-HSA-1660516 | 31 | 226 |
| Signaling by NTRK2 (TRKB) | R-HSA-9006115 | 31 | 231 |
| A tetrasaccharide linker sequence is required for GAG synthesis | R-HSA-1971475 | 31 | 250 |
| Activation of G protein gated Potassium channels | R-HSA-1296041 | 31 | 461 |
| RUNX2 regulates osteoblast differentiation | R-HSA-8940973 | 30 | 96 |
| TRAF6 mediated IRF7 activation | R-HSA-933541 | 30 | 163 |
| Regulation of pyruvate dehydrogenase (PDH) complex | R-HSA-204174 | 30 | 238 |
| Norepinephrine Neurotransmitter Release Cycle | R-HSA-181430 | 30 | 253 |
| Sema4D in semaphorin signaling | R-HSA-400685 | 30 | 256 |
| SHC1 events in ERBB2 signaling | R-HSA-1250196 | 30 | 274 |
| Recognition of DNA damage by PCNA-containing replication complex | R-HSA-110314 | 30 | 408 |
| FRS-mediated FGFR2 signaling | R-HSA-5654700 | 30 | 420 |
| Signaling by FGFR3 point mutants in cancer | R-HSA-8853338 | 30 | 424 |
| Resolution of D-loop Structures through Synthesis-Dependent Strand Annealing (SDSA) | R-HSA-5693554 | 30 | 425 |
| Signaling by FGFR3 in disease | R-HSA-5655332 | 30 | 432 |
| CD209 (DC-SIGN) signaling | R-HSA-5621575 | 29 | 119 |
| The role of Nef in HIV-1 replication and disease pathogenesis | R-HSA-164952 | 29 | 134 |
| PIWI-interacting RNA (piRNA) biogenesis | R-HSA-5601884 | 29 | 150 |
| TRAF6 mediated NF-kB activation | R-HSA-933542 | 29 | 201 |
| G0 and Early G1 | R-HSA-1538133 | 29 | 204 |
| Long-term potentiation | R-HSA-9620244 | 29 | 428 |
| EGR2 and SOX10-mediated initiation of Schwann cell myelination | R-HSA-9619665 | 28 | 70 |
| HSF1-dependent transactivation | R-HSA-3371571 | 28 | 111 |
| WNT ligand biogenesis and trafficking | R-HSA-3238698 | 28 | 127 |
| G1/S-Specific Transcription | R-HSA-69205 | 28 | 128 |
| LDL clearance | R-HSA-8964038 | 28 | 132 |
| NOTCH3 Activation and Transmission of Signal to the Nucleus | R-HSA-9013507 | 28 | 147 |
| Signaling by FLT3 fusion proteins | R-HSA-9703465 | 28 | 177 |
| Termination of O-glycan biosynthesis | R-HSA-977068 | 28 | 197 |
| Deadenylation of mRNA | R-HSA-429947 | 28 | 283 |
| Downregulation of TGF-beta receptor signaling | R-HSA-2173788 | 28 | 296 |
| SHC-mediated cascade:FGFR2 | R-HSA-5654699 | 28 | 320 |
| FRS-mediated FGFR1 signaling | R-HSA-5654693 | 28 | 347 |
| mTORC1-mediated signalling | R-HSA-166208 | 28 | 352 |
| Dopamine Neurotransmitter Release Cycle | R-HSA-212676 | 28 | 494 |
| Metabolism of Angiotensinogen to Angiotensins | R-HSA-2022377 | 27 | 69 |
| Constitutive Signaling by AKT1 E17K in Cancer | R-HSA-5674400 | 27 | 105 |
| ATF4 activates genes in response to endoplasmic reticulum stress | R-HSA-380994 | 27 | 109 |
| Interleukin-7 signaling | R-HSA-1266695 | 27 | 118 |
| RAF-independent MAPK1/3 activation | R-HSA-112409 | 27 | 139 |
| Cellular hexose transport | R-HSA-189200 | 27 | 145 |
| Mitochondrial calcium ion transport | R-HSA-8949215 | 27 | 163 |
| Downregulation of SMAD2/3:SMAD4 transcriptional activity | R-HSA-2173795 | 27 | 174 |
| Regulation of IFNA signaling | R-HSA-912694 | 27 | 230 |
| RHO GTPases activate PAKs | R-HSA-5627123 | 27 | 239 |
| Gap-filling DNA repair synthesis and ligation in GG-NER | R-HSA-5696397 | 27 | 325 |
| FRS-mediated FGFR4 signaling | R-HSA-5654712 | 27 | 360 |
| Unblocking of NMDA receptors, glutamate binding and activation | R-HSA-438066 | 27 | 434 |
| Estrogen-dependent nuclear events downstream of ESR-membrane signaling | R-HSA-9634638 | 26 | 63 |
| Initiation of Nuclear Envelope (NE) Reformation | R-HSA-2995383 | 26 | 65 |
| Glycogen breakdown (glycogenolysis) | R-HSA-70221 | 26 | 105 |
| ERK/MAPK targets | R-HSA-198753 | 26 | 113 |
| Deregulated CDK5 triggers multiple neurodegenerative pathways in Alzheimer's disease models | R-HSA-8862803 | 26 | 118 |
| Neurodegenerative Diseases | R-HSA-8863678 | 26 | 118 |
| Intrinsic Pathway of Fibrin Clot Formation | R-HSA-140837 | 26 | 156 |
| Glycogen synthesis | R-HSA-3322077 | 26 | 159 |
| Signaling by ERBB2 TMD/JMD mutants | R-HSA-9665686 | 26 | 241 |
| Acetylcholine Neurotransmitter Release Cycle | R-HSA-264642 | 26 | 242 |
| SHC-mediated cascade:FGFR1 | R-HSA-5654688 | 26 | 289 |
| TNFR1-induced NFkappaB signaling pathway | R-HSA-5357956 | 26 | 303 |
| Ras activation upon Ca2+ influx through NMDA receptor | R-HSA-442982 | 26 | 353 |
| G-protein activation | R-HSA-202040 | 26 | 411 |
| APC-Cdc20 mediated degradation of Nek2A | R-HSA-179409 | 26 | 432 |
| FGFR2 alternative splicing | R-HSA-6803529 | 26 | 628 |
| Assembly of active LPL and LIPC lipase complexes | R-HSA-8963889 | 25 | 52 |
| NOTCH3 Intracellular Domain Regulates Transcription | R-HSA-9013508 | 25 | 140 |
| Signalling to RAS | R-HSA-167044 | 25 | 155 |
| Chondroitin sulfate biosynthesis | R-HSA-2022870 | 25 | 158 |
| Common Pathway of Fibrin Clot Formation | R-HSA-140875 | 25 | 171 |
| Constitutive Signaling by Ligand-Responsive EGFR Cancer Variants | R-HSA-1236382 | 25 | 206 |
| Signaling by Ligand-Responsive EGFR Variants in Cancer | R-HSA-5637815 | 25 | 206 |
| SHC-mediated cascade:FGFR4 | R-HSA-5654719 | 25 | 272 |
| CTLA4 inhibitory signaling | R-HSA-389513 | 25 | 278 |
| FRS-mediated FGFR3 signaling | R-HSA-5654706 | 25 | 320 |
| Insertion of tail-anchored proteins into the endoplasmic reticulum membrane | R-HSA-9609523 | 25 | 324 |
| Inactivation of CSF3 (G-CSF) signaling | R-HSA-9705462 | 25 | 354 |
| VxPx cargo-targeting to cilium | R-HSA-5620916 | 25 | 388 |
| Synthesis, secretion, and deacylation of Ghrelin | R-HSA-422085 | 24 | 46 |
| Transcriptional regulation of pluripotent stem cells | R-HSA-452723 | 24 | 104 |
| Rap1 signalling | R-HSA-392517 | 24 | 107 |
| STING mediated induction of host immune responses | R-HSA-1834941 | 24 | 140 |
| RORA activates gene expression | R-HSA-1368082 | 24 | 150 |
| Sema4D induced cell migration and growth-cone collapse | R-HSA-416572 | 24 | 191 |
| IKK complex recruitment mediated by RIP1 | R-HSA-937041 | 24 | 192 |
| RHO GTPases Activate ROCKs | R-HSA-5627117 | 24 | 204 |
| BBSome-mediated cargo-targeting to cilium | R-HSA-5620922 | 24 | 305 |
| Translesion Synthesis by POLH | R-HSA-110320 | 24 | 332 |
| APC/C:Cdc20 mediated degradation of Cyclin B | R-HSA-174048 | 24 | 401 |
| Regulation of gene expression in beta cells | R-HSA-210745 | 23 | 30 |
| TP53 Regulates Transcription of Genes Involved in Cytochrome C Release | R-HSA-6803204 | 23 | 42 |
| Interleukin-37 signaling | R-HSA-9008059 | 23 | 55 |
| Regulation of TP53 Activity through Methylation | R-HSA-6804760 | 23 | 72 |
| Gastrin-CREB signalling pathway via PKC and MAPK | R-HSA-881907 | 23 | 81 |
| Apoptotic factor-mediated response | R-HSA-111471 | 23 | 102 |
| Pre-NOTCH Processing in Golgi | R-HSA-1912420 | 23 | 117 |
| NOTCH2 Activation and Transmission of Signal to the Nucleus | R-HSA-2979096 | 23 | 135 |
| ZBP1(DAI) mediated induction of type I IFNs | R-HSA-1606322 | 23 | 142 |
| activated TAK1 mediates p38 MAPK activation | R-HSA-450302 | 23 | 162 |
| Signaling by NODAL | R-HSA-1181150 | 23 | 169 |
| Presynaptic function of Kainate receptors | R-HSA-500657 | 23 | 234 |
| SHC-mediated cascade:FGFR3 | R-HSA-5654704 | 23 | 240 |
| RNA Polymerase III Transcription Termination | R-HSA-73980 | 23 | 242 |
| GAB1 signalosome | R-HSA-180292 | 23 | 252 |
| Ephrin signaling | R-HSA-3928664 | 23 | 303 |
| Trafficking of GluR2-containing AMPA receptors | R-HSA-416993 | 23 | 337 |
| Serotonin Neurotransmitter Release Cycle | R-HSA-181429 | 23 | 342 |
| Defective B3GAT3 causes JDSSDHD | R-HSA-3560801 | 22 | 41 |
| PINK1-PRKN Mediated Mitophagy | R-HSA-5205685 | 22 | 103 |
| Chaperone Mediated Autophagy | R-HSA-9613829 | 22 | 112 |
| Nef-mediates down modulation of cell surface receptors by recruiting them to clathrin adapters | R-HSA-164938 | 22 | 122 |
| Depolymerisation of the Nuclear Lamina | R-HSA-4419969 | 22 | 124 |
| JNK (c-Jun kinases) phosphorylation and activation mediated by activated human TAK1 | R-HSA-450321 | 22 | 145 |
| RHO GTPases activate CIT | R-HSA-5625900 | 22 | 154 |
| Regulation of FZD by ubiquitination | R-HSA-4641263 | 22 | 233 |
| Defective B4GALT7 causes EDS, progeroid type | R-HSA-3560783 | 21 | 20 |
| Defective B3GALT6 causes EDSP2 and SEMDJL1 | R-HSA-4420332 | 21 | 20 |
| Defective C1GALT1C1 causes Tn polyagglutination syndrome (TNPS) | R-HSA-5083632 | 21 | 39 |
| mRNA decay by 5' to 3' exoribonuclease | R-HSA-430039 | 21 | 169 |
| Defective GALNT3 causes familial hyperphosphatemic tumoral calcinosis (HFTC) | R-HSA-5083625 | 20 | 19 |
| Defects of Formation of Fibrin Clot (Clotting Cascade) | R-HSA-9651496 | 20 | 45 |
| Diseases of hemostasis | R-HSA-9671793 | 20 | 45 |
| SUMOylation of transcription factors | R-HSA-3232118 | 20 | 58 |
| NOTCH4 Intracellular Domain Regulates Transcription | R-HSA-9013695 | 20 | 102 |
| MET activates PTK2 signaling | R-HSA-8874081 | 20 | 140 |
| Signaling by FGFR2 IIIa TM | R-HSA-8851708 | 20 | 141 |
| Phase 4 - resting membrane potential | R-HSA-5576886 | 20 | 158 |
| Phosphorylation of the APC/C | R-HSA-176412 | 20 | 188 |
| Activation of IRF3/IRF7 mediated by TBK1/IKK epsilon | R-HSA-936964 | 20 | 235 |
| Spry regulation of FGF signaling | R-HSA-1295596 | 19 | 82 |
| TICAM1, RIP1-mediated IKK complex recruitment | R-HSA-168927 | 19 | 84 |
| RIP-mediated NFkB activation via ZBP1 | R-HSA-1810476 | 19 | 112 |
| EPHA-mediated growth cone collapse | R-HSA-3928663 | 19 | 122 |
| Ligand-dependent caspase activation | R-HSA-140534 | 19 | 127 |
| Transcription of E2F targets under negative control by DREAM complex | R-HSA-1362277 | 19 | 128 |
| FGFR1 ligand binding and activation | R-HSA-190242 | 19 | 203 |
| Beta-catenin phosphorylation cascade | R-HSA-196299 | 19 | 206 |
| Response of EIF2AK1 (HRI) to heme deficiency | R-HSA-9648895 | 18 | 60 |
| Polo-like kinase mediated events | R-HSA-156711 | 18 | 68 |
| NRIF signals cell death from the nucleus | R-HSA-205043 | 18 | 89 |
| Zinc transporters | R-HSA-435354 | 18 | 125 |
| MAP3K8 (TPL2)-dependent MAPK1/3 activation | R-HSA-5684264 | 18 | 125 |
| GRB2 events in ERBB2 signaling | R-HSA-1963640 | 18 | 138 |
| CRMPs in Sema3A signaling | R-HSA-399956 | 18 | 280 |
| MECP2 regulates neuronal receptors and channels | R-HSA-9022699 | 17 | 26 |
| FOXO-mediated transcription of cell cycle genes | R-HSA-9617828 | 17 | 57 |
| Aberrant regulation of mitotic G1/S transition in cancer due to RB1 defects | R-HSA-9659787 | 17 | 95 |
| Defective binding of RB1 mutants to E2F1,(E2F2, E2F3) | R-HSA-9661069 | 17 | 95 |
| FOXO-mediated transcription of cell death genes | R-HSA-9614657 | 16 | 48 |
| Regulation of gene expression in late stage (branching morphogenesis) pancreatic bud precursor cells | R-HSA-210744 | 16 | 63 |
| Formation of Senescence-Associated Heterochromatin Foci (SAHF) | R-HSA-2559584 | 16 | 76 |
| SUMOylation of DNA methylation proteins | R-HSA-4655427 | 16 | 88 |
| Transcription of E2F targets under negative control by p107 (RBL1) and p130 (RBL2) in complex with HDAC1 | R-HSA-1362300 | 16 | 90 |
| Signaling by NOTCH1 HD Domain Mutants in Cancer | R-HSA-2691230 | 16 | 132 |
