# Supplementary material for: Risk stratification and pathway analysis based on graph neural network and interpretable algorithm
Source: BMC Bioinformatics. 2022 Sep 27;23:394. doi: 10.1186/s12859-022-04950-1 (PMC9516820; doi:10.1186/s12859-022-04950-1)
Supplement: Supplementary file 2 — Additional file 2. The architecture of DNN and PGDNN. [file 12859_2022_4950_MOESM2_ESM.pdf]

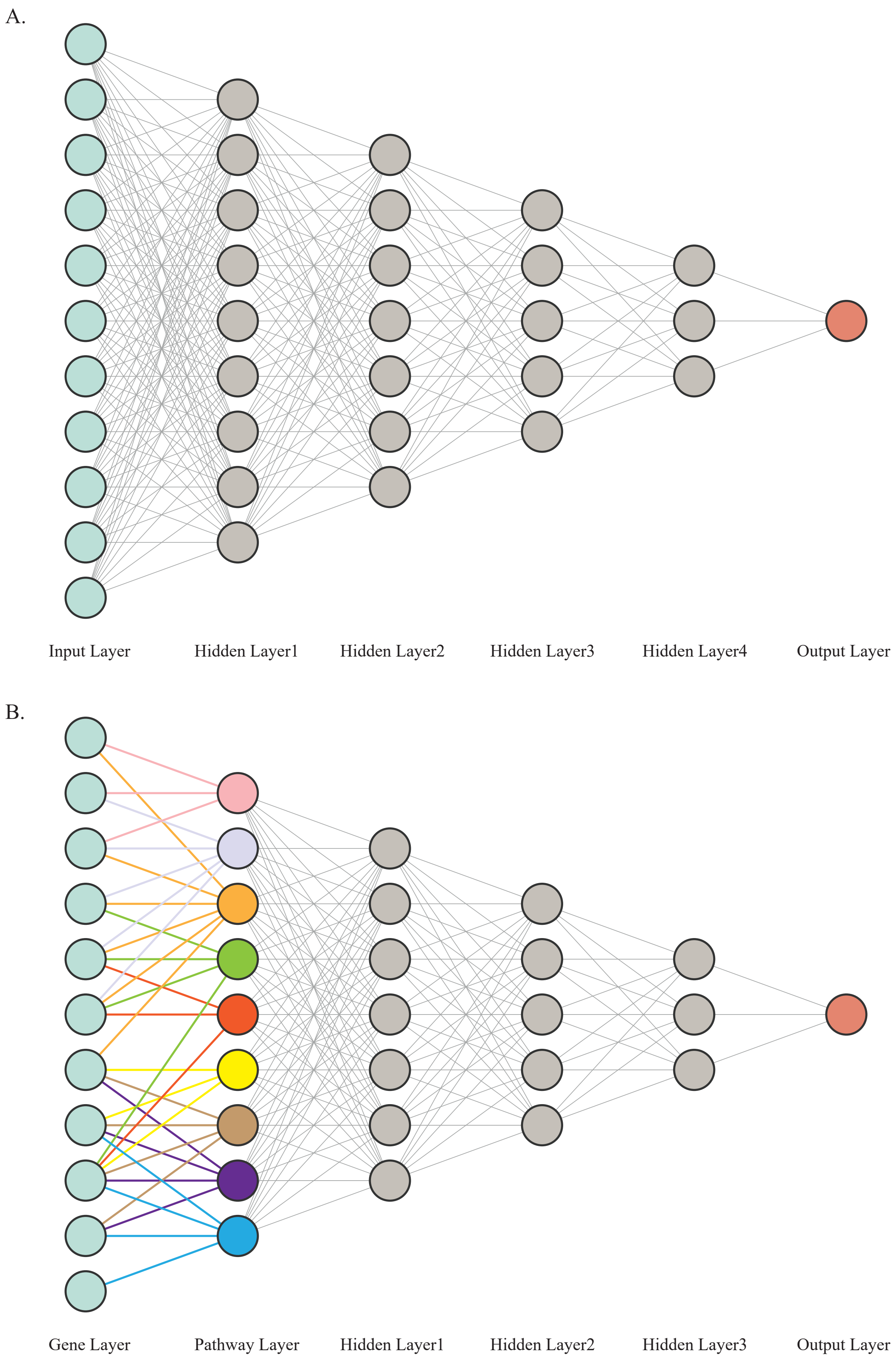

Additional file 2. The architecture of DNN and PGDNN. A) The DNN comprised of one input layer, four hidden layers and one output layer. The nodes number of hidden layers were 855, 128, 64 and 16, respectively. ReLU was used as an activation function between layers and a sigmoid function was applied in the output layer. B) the architecture of PGDNN was same with DNN, but the first hidden layer, named pathway layer, which represents the biological pathways linked with input genes.
