# Supplementary material for: Risk stratification and pathway analysis based on graph neural network and interpretable algorithm
Source: BMC Bioinformatics. 2022 Sep 27;23:394. doi: 10.1186/s12859-022-04950-1 (PMC9516820; doi:10.1186/s12859-022-04950-1)
Supplement: Supplementary file 3 — Additional file 3. The key pathways associated with cancer survival identified by PathGNN. [file 12859_2022_4950_MOESM3_ESM.docx]

**Additional file 3** | The key pathways associated with cancer survival identified by PathGNN. "Score" indicates the median value of all patients' IG score in the pathway; "Score^z^ " indicates the z-transform value for IG score and represents correlation with clinical outcome. "Pvalue" represents the statistical difference between two groups that dichotomized by a median split of IG score in the specific pathways. "Score" indicates

| **Pathway** | **Cancer** | **Score** | **Score^z^** | **Pvalue** | **Ref** |
| --- | --- | --- | --- | --- | --- |
| Aberrant regulation of mitotic G1/S transition in cancer due to RB1 defects | KIRC | 4.77E-16 | 3.523 | 7.86E-14 | [1] |
| Sialic acid metabolism | KIRC | 3.07E-16 | 2.305 | 7.86E-14 | [2] |
| Iron uptake and transport | KIRC | 2.24E-16 | 2.286 | 7.18E-14 | - |
| Signaling by FGFR | KIRC | 2.77E-16 | 3.525 | 1.93E-14 | [3] |
| Stabilization of p53 | KIRC | 2.68E-16 | 1.97 | 7.67E-14 | [4] |
| Aberrant regulation of mitotic G1/S transition in cancer due to RB1 defects | LGG | 4.85E-15 | 4.955 | 2.69E-05 | [5, 6] |
| Formation of Senescence-Associated Heterochromatin Foci (SAHF) | LGG | 7.42E-15 | 6.187 | 3.23E-05 | - |
| Extra-nuclear estrogen signaling | LGG | 5.25E-15 | 4.038 | 1.14E-04 | - |
| L1CAM interactions | LGG | 3.66E-15 | 2.668 | 4.57E-05 | [7] |
| MECP2 regulates neuronal receptors and channels | LGG | 2.97E-15 | 2.293 | 1.01E-04 | [8] |
| Nucleobase biosynthesis | LGG | 2.79E-15 | 2.132 | 2.09E-06 | - |
| Regulation of TP53 Expression and Degradation | LGG | 3.85E-15 | 3.927 | 4.07E-05 | [9] |
| Switching of origins to a post-replicative state | LGG | 2.73E-15 | 2.447 | 8.03E-06 | - |
| Zinc transporters | LGG | 2.82E-15 | 2.134 | 6.89E-06 | - |
| Aberrant regulation of mitotic cell cycle due to RB1 defects | LUAD | 8.09E-18 | 2.161 | 2.30E-09 | [5, 6] |
| Aberrant regulation of mitotic G1/S transition in cancer due to RB1 defects | LUAD | 1.08E-17 | 3.153 | 1.82E-09 | [5, 6] |
| Formation of Senescence-Associated Heterochromatin Foci (SAHF) | LUAD | 8.45E-18 | 2.589 | 1.12E-09 | - |
| Sialic acid metabolism | LUAD | 1.82E-17 | 5.14 | 1.63E-09 | [2] |
| Amino acids regulate mTORC1 | LUAD | 1.53E-17 | 3.855 | 1.96E-09 | [10] |
| Assembly of collagen fibrils and other multimeric structures | LUAD | 5.62E-18 | 1.905 | 7.14E-10 | - |
| Asymmetric localization of PCP proteins | LUAD | 4.36E-18 | 1.952 | 1.18E-09 | - |
| B-WICH complex positively regulates rRNA expression | LUAD | 1.95E-17 | 5.007 | 1.02E-08 | - |
| E3 ubiquitin ligases ubiquitinate target proteins | LUAD | 1.43E-17 | 3.1 | 1.15E-09 | [11] |
| Metalloprotease DUBs | LUAD | 5.34E-18 | 2.104 | 2.24E-09 | - |
| Platelet activation, signaling and aggregation | LUAD | 1.09E-17 | 3.866 | 5.89E-09 | [12] |
| Regulation of RUNX3 expression and activity | LUAD | 1.38E-17 | 2.372 | 1.59E-09 | [13] |
| rRNA processing in the nucleus and cytosol | LUAD | 5.96E-18 | 1.928 | 2.77E-10 | - |
| RUNX1 regulates transcription of genes involved in differentiation of HSCs | SKCM | 1.25E-15 | 2.095 | 6.09E-04 | [14] |
| Metabolism of cofactors | SKCM | 1.15E-15 | 2.029 | 8.68E-02 | - |
| Muscle contraction | SKCM | 1.19E-15 | 2.272 | 3.15E-02 | - |
| Signaling by ERBB4 | SKCM | 2.13E-15 | 3.628 | 1.54E-02 | [15] |

**Reference**

1. Bai Q, Liu L, Xia Y, Long Q, Wang J, Xu J, et al. Clinical characteristics and prognosis of osteosarcoma in young children: a retrospective series of 15 cases. 2011. https://doi.org/10.1186/s12885-015-1906-5.

2. Cornelissen LAM, Blanas A, van der Horst JC, Kruijssen L, Zaal A, O’toole T, et al. Disruption of sialic acid metabolism drives tumor growth by augmenting CD8 + T cell apoptosis. 2018. https://doi.org/10.1002/ijc.32084.

3. Massari F, Ciccarese C, Santoni M, Lopez-Beltran A, Scarpelli M, Montironi R, et al. Targeting fibroblast growth factor receptor (FGFR) pathway in renal cell carcinoma. Expert Review of Anticancer Therapy. 2015;15.

4. Lee SH, Lee WK, Kim N, Kang JH, Kim KH, Kim SG, et al. Renal cell carcinoma is abrogated by p53 stabilization through transglutaminase 2 inhibition. Cancers (Basel). 2018;10.

5. Linn P, Kohno S, Sheng J, Kulathunga N, Yu H, Zhang Z, et al. Targeting RB1 loss in cancers. Cancers. 2021;13.

6. Oser MG, Fonseca R, Chakraborty AA, Brough R, Spektor A, Jennings RB, et al. Cells lacking the RB1 tumor suppressor gene are hyperdependent on aurora B kinase for survival. Cancer Discov. 2019;9.

7. Er EE, Valiente M, Ganesh K, Zou Y, Agrawal S, Hu J, et al. Pericyte-like spreading by disseminated cancer cells activates YAP and MRTF for metastatic colonization. Nat Cell Biol. 2018;20.

8. Chahrour M, Sung YJ, Shaw C, Zhou X, Wong STC, Qin J, et al. MeCP2, a key contributor to neurological disease, activates and represses transcription. Science (1979). 2008;320.

9. Noor H, Briggs NE, McDonald KL, Holst J, Vittorio O. Tp53 mutation is a prognostic factor in lower grade glioma and may influence chemotherapy efficacy. Cancers (Basel). 2021;13.

10. Zhang Q, Zhang Y, Chen Y, Qian J, Zhang X, Yu K. A Novel mTORC1/2 Inhibitor (MTI-31) Inhibits Tumor Growth, Epithelial-Mesenchymal Transition, Metastases, and Improves Antitumor Immunity in Preclinical Models of Lung Cancer. https://doi.org/10.1158/1078-0432.CCR-18-2548.

11. Li K, Zheng X, Tang H, Zang YS, Zeng C, Liu X, et al. E3 ligase mkrn3 is a tumor suppressor regulating pabpc1 ubiquitination in non–small cell lung cancer. Journal of Experimental Medicine. 2021;218.

12. Shi L, Li Y, Yu T, Wang Z, Zhou C, Xing W, et al. Predictable resistance and overall survival of gemcitabine/cisplatin by platelet activation index in non-small cell lung cancer. Medical Science Monitor. 2018;24.

13. Lee YS, Lee JW, Jang JW, Chi XZ, Kim JH, Li YH, et al. Runx3 Inactivation Is a Crucial Early Event in the Development of Lung Adenocarcinoma. Cancer Cell. 2013;24.

14. Scheitz CJF, Lee TS, McDermitt DJ, Tumbar T. Defining a tissue stem cell-driven Runx1/Stat3 signalling axis in epithelial cancer. EMBO Journal. 2012;31.

15. Settleman J. A Therapeutic Opportunity in Melanoma: ErbB4 Makes a Mark on Skin. Cancer Cell. 2009;16.
